# Comprehensive analysis of PSMD family members and validation of PSMD9 as a potential therapeutic target in human glioblastoma

**DOI:** 10.1111/cns.14366

**Published:** 2023-07-23

**Authors:** Yaquan Li, Xuemeng Liu, Feihu Zhao, Zhimin Zhao, Xingang Li, Jian Wang, Bin Huang, Anjing Chen

**Affiliations:** ^1^ Department of Neurosurgery Qilu Hospital Cheeloo College of Medicine and Institute of Brain and Brain‐Inspired Science Shandong University Jinan China; ^2^ Jinan Microecological Biomedicine Shandong Laboratory and Shandong Key Laboratory of Brain Function Remodeling Jinan China; ^3^ Department of Biomedicine University of Bergen Bergen Norway

**Keywords:** glioblastoma, panobinostat, prognosis, PSMD family, PSMD9

## Abstract

**Aims:**

PSMD family members, as important components of the 26S proteasome, are well known to be involved in protein degradation. However, their role in glioblastoma (GBM) has not been rigorously investigated. We aimed to perform systematic analysis of the expression signature, prognostic significance and functions of PSMD family genes in GBM to reveal potential prognostic markers and new therapeutic targets among PSMD family members.

**Methods:**

In this study, we systemically analyzed PSMD family members in terms of their expression profiles, prognostic implications, DNA methylation levels, and genetic alterations; the relationships between their expression levels and immune infiltration and drug sensitivity; and their potential functional enrichment in GBM through bioinformatics assessment. Moreover, in vitro and in vivo experiments were used to validate the biological functions of PSMD9 and its targeted therapeutic effect in GBM.

**Results:**

The mRNA levels of PSMD5/8/9/10/11/13/14 were higher in GBM than in normal brain tissues, and the mRNA levels of PSMD1/4/5/8/9/11/12 were higher in high‐grade glioma (WHO grade III & IV) than in low‐grade glioma (WHO grade II). High mRNA expression of PSMD2/6/8/9/12/13/14 and low mRNA expression of PSMD7 were associated with poor overall survival (OS). Multivariate Cox regression analysis identified PSMD2/5/6/8/9/10/11/12 as independent prognostic factors for OS prediction. In addition, the protein–protein interaction network and gene set enrichment analysis results suggested that PSMD family members and their interacting molecules were involved in the regulation of the cell cycle, cell invasion and migration, and other biological processes in GBM. In addition, knockdown of PSMD9 inhibited cell proliferation, invasion and migration and induced G2/M cell cycle arrest in LN229 and A172 GBM cells. Moreover, PSMD9 promoted the malignant progression of GBM in vivo. GBM cell lines with high PSMD9 expression were more resistant to panobinostat, a potent deacetylase inhibitor, than those with low PSMD9 expression. In vitro and in vivo experiments further validated that PSMD9 overexpression rescued the GBM inhibitory effect of panobinostat.

**Conclusion:**

This study provides new insights into the value of the PSMD family in human GBM diagnosis and prognosis evaluation, and we further identified PSMD9 as a potential therapeutic target. These findings may lead to the development of effective therapeutic strategies for GBM.

## Graphical abstract

Comprehensive analysis of the value of PSMD family members in glioblastoma diagnosis and prognosis evaluation identified PSMD9 as a potential therapeutic target.

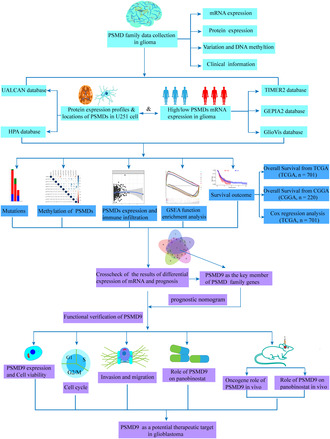

## 1 INTRODUCTION

Glioblastoma (GBM) is the most common and aggressive intracranial tumor in adults. For many years, conventional treatment strategies for most GBM cases, including surgery, radiation and chemotherapy, have showed limited efficacy, with little improvement in median survival time.^1^ Although significant efforts have been made to identify functional therapeutic targets for GBM to control its growth and recurrence, novel biomarkers are needed as prognostic indicators to guide individualized treatment and improve GBM patient prognosis.

In recent studies, it has been shown that dysfunction of the ubiquitin–proteasome system (UPS) is associated with the development and progression of various cancers. In this regard, ubiquitination causes activation or deactivation of tumorigenic pathways, and numerous UPS enzymes (E1, E2, E3) have been reported to have anti‐ or protumoral roles in GBM. In the siRNA screening analysis that revealed relevant genes for GBM survival, 22% (12/55) were components of the 20S and 26S proteasome subunits.^2^, ^3^ Deubiquitinases (DUBs), another component of the UPS, are dysregulated in multiple cancers, including GBM. This indicates that DUB dysfunction is closely related to the oncogenesis of glioma, and it has potential clinical significance to treat GBM by targeting DUBs.^1^, ^2^ Numerous natural and synthetic compounds, including classic proteasome inhibitors, have been used to treat glioma by targeting the UPS.^2^

The UPS is the major proteolytic system that controls protein degradation in eukaryotic cells and regulates many cellular processes, such as DNA repair, stress response and cell proliferation.^4^ The UPS consists of specific enzymes that modify protein substrates with ubiquitin and proteasomes responsible for the proteolysis of ubiquitin‐tagged substrates.^5^ The proteasome, as the endpoint of the ubiquitin–proteasome system, is the main proteolytic machinery responsible for regulating protein degradation in eukaryotic cells.^6^ One type of proteosome, the 26S proteasome, consists of a cylindrical 20S complex and one or two regulatory 19S complexes.^7^, ^8^ The 20S core is constructed from inner α‐rings and outer β‐rings, which are both divided into 7 structurally similar subunits: proteasome 20S subunit α (PSMA1–7) and β (PSMB1–7). The 19S cap complex is composed of a base and a lid subcomplex, which are further categorized into ATPase subunits (PSMC1–6) and non‐ATPase subunits (PSMD1–14).^9^

The PSMD family consists of 14 members, namely, PSMD1–14, which play an important role in regulating the 26S proteasome. A previous study showed that PSMD1 and PSMD3 were plausible therapeutic targets worthy of future investigation in chronic myeloid leukemia.^10^ PSMD2 knockdown inhibited breast cancer cell proliferation and arrested the cell cycle at the G0/G1 phase.^11^ PSMD6 and PSMD11 could serve as potential prognostic and diagnostic biomarkers for patients with early‐stage pancreatic ductal adenocarcinoma after pancreaticoduodenectomy.^12^ Targeting PSMD10 may be a strategy in hepatocellular carcinoma treatment, as this approach would suppress autophagy.^13^ PSMD14 is overexpressed in ovarian cancer tissues and is a biomarker and therapeutic candidate for ovarian cancer.^14^ It is clear that many up‐ and downregulated genes of the PSMD family are associated with oncogenes or oncogenic functions during cancer development. However, the role of PSMD family members in the development of GBM is not fully understood, and their prognostic value for GBM is still unknown.

Therefore, in this study, we used a variety of available public databases to systematically analyze the expression characteristics of the PSMD family, the relationships with clinical prognosis and the underlying molecular mechanisms in GBM. Then, in vitro and in vivo experiments were used to further validate the specific results of the above analysis with the goal of finding new targets for the treatment of GBM as well as therapeutic strategies.

## 2 MATERIALS AND METHODS

### 2.1 Glioma tissue specimen collection

Twenty surgically removed glioma tissue specimens were collected from patients in the Department of Neurosurgery of Qilu Hospital of Shandong University from May 2022 to September 2022. All patients had a pathologically confirmed glioma diagnosis and signed an informed consent form, and the study was approved by the ethics committee.

### 2.2 Expression data acquisition of PSMD family members

Data on the differential mRNA expression of PSMD in GBM and normal brain tissues were obtained from the TIMER2 (http://timer.comp‐genomics.org/) and GEPIA2 (http://gepia2.cancer‐pku.cn/#index) databases, and the expression in different grades of glioma was obtained from the GLIOVIS database (http://gliovis.bioinfo.cnio.es/). The TIMER2 database allows users to study associations between gene expression and tumor features in TCGA. GEPIA2 is an interactive web application for gene expression analysis based on RNA sequencing expression data of 9736 tumors and 8587 normal samples from the TCGA and Genotype‐Tissue Expression (GTEx) databases. The GlioVis data portal can be used to analyze gene expression in gliomas of different grades in the CGGA database. Proteomic data were downloaded from the UALCAN (The University of Alabama at Birmingham Cancer) data analysis portal. UALCAN provides a protein expression analysis option using data from the Clinical Proteomic Tumor Analysis Consortium (CPTAC) dataset. Immunohistochemistry images of the PSMD family were obtained from the Human Protein Atlas (HPA) database (https://www.proteinatlas.org/). Immunocytochemistry images were obtained to detect and visualize PSMD proteins in the GBM U251 cell line.

### 2.3 Survival analysis

The RNA sequencing data and clinical information in the TCGA‐GBM dataset were downloaded from UCSC Xena (http://xena.ucsc.edu/; GBM samples, *n* = 701).^15^ The RNA sequencing data and clinical information in the CGGA dataset were obtained from the official website (http://www.cgga.org.cn/index.jsp; GBM samples, *n* = 220).^16^ Patients were divided into high and low expression groups based on the median gene expression to analyze the overall survival (OS) of GBM patients. The “survival” package of R studio software was used for statistical analysis, and the “survminer” package was used for Kaplan–Meier curves and visualization. Multifactorial regression analysis was used to identify independent prognostic factors for survival time.

### 2.4 PSMD gene methylation analysis

The data on the correlations between PSMD methylation and mRNA expression and methylation sites were obtained from the TCGA. The GSCA platform (http://bioinfo.life.hust.edu.cn/GSCA/#/) was used to analyze the association between paired mRNA expression and methylation. The “corrplot” R package was used to investigate the correlations between methylation sites and PSMD gene expression.

### 2.5 Analysis of genetic alterations and tumor‐infiltrating immune cells

We analyzed the genomic alteration profiles, including mutation frequency and single nucleotide variants (SNVs), of PSMD members in GBM patients with cBioPortal (www.cbioportal.org) dataset analysis and the GSCA platform. We then downloaded data on tumor‐infiltrating immune cells from the TIMER2 database to analyze the correlations between the expression levels of PSMD members and the abundance of GBM‐infiltrating immune cells. Correlations between PSMD levels and the sensitivity to drugs in the Genomics of Therapeutics Response Portal (top 30) in GBM were obtained from the GSCA platform.

### 2.6 Identification of protein interactions and relevant signaling pathways

The package “corrplot” of R studio software and the STRING database (http://string‐db.org) were used to study the interaction relationships between PSMD family members. The Perl and “ggplot2” package were used to perform gene set enrichment analysis (GSEA). GeneMANIA (http://genemania.org/) and GSCA were used to analyze gene–gene interactions and related cancer pathway activity.

### 2.7 Construction and validation of a prognostic nomogram for PSMD9

To establish a prognostic model for predicting OS in GBM, a nomogram based on the expression level of PSMD9, patient age, patient gender and tumor grade in the TCGA database was constructed. Then, time‐dependent receiver operating characteristic curves (ROCs) were constructed to evaluate the predictive value of PSMD9 for OS. Calibration curves of the 1‐, 3‐, and 5‐year survival rates were drawn to verify the consistency of the OS data. The “survival”, “survminer”, “timeROC”, “regplot” and “rms” packages of R software were used.

### 2.8 Cell culture and transient transfection

The human GBM cell lines LN229, U118, U251 and A172 were obtained from the Culture Collection of the Chinese Academy of Sciences (Shanghai, China). Normal human astrocytes (NHAs) and primary GBM#P3 and GBM#BG5 cells were kind gifts from Professor Rolf Bjerkvig, University of Bergen (Norway). All cell lines and NHAs were cultured in Dulbecco's modified Eagle's medium (DMEM; Life Technologies, USA) supplemented with 10% fetal bovine serum (FBS; VivaCell, Shanghai, China). All primary GBM cells were cultured in neurobasal medium. Transient transfections were performed for siRNAs with 4 μL of Lipofectamine 2000 (Thermo Fisher Scientific) and 5 μL of siRNA, and plasmids were added to 6‐well plates using a ratio of 2 μg:5 μL of plasmid to transfection reagent per well. The sequences of siRNAs were as follows: si‐negative control: 5′‐TTCTCCGAACGTGTCACGT‐3′; si‐PSMD9‐1: 5′‐GCAAGUGGAUGAU GAGAUUTT‐3′; si‐PSMD9‐2: 5′‐GACGAGGAAGCGA GGCAGATT‐3′; si‐PSMD 9–3: 5′‐ CAACAUUAUUCCUCUGCAATT‐3′. The transient plasmid for PSMD9 was pcDNA3.1(+)‐PSMD9‐3xFLAG (OBiO Technology, Shanghai, China).

### 2.9 Lentiviral transduction

Lentiviral vectors expressing human short hairpin RNA (shRNA) targeting PSMD9 (shPSMD9, GenePharma Inc, Shanghai, China) or scrambled control (shNC) were used to generate stable cell clones expressing shPSMD9 or a nonspecific shRNA as the control. The sequence of the shRNA used was as follows: GCAAGUGGAUGAU GAGAUUTT. Lentiviral vectors expressing human mRNA targeting PSMD9 (GenePharma Inc., Shanghai, China) or scrambled control (negative control) were used to generate stable cell clones overexpressing PSMD9 or a nonspecific RNA as the control. Clones infected with lentivirus were selected using 1 mg/mL puromycin (#HY‐K1057, MedChemExpress; USA).

### 2.10 Immunohistochemistry (IHC)

Glioma samples were obtained from 20 patients (WHO II, *n* = 6; WHO III, *n* = 6; and WHO IV, *n* = 8) who had undergone surgeries performed at the Department of Neurosurgery at Qilu Hospital. Tissues were fixed with 4% formalin, embedded in paraffin, and sectioned. IHC assays were performed using the SPlink Detection Kit and DAB (ZSGB‐BIO; Beijing, China) according to the manufacturer's instructions. The following primary antibodies were used: PSMD9 (#67338‐1‐Ig, 1:400; ProteinTech, Chicago, USA) and Ki67 (#GB111499, 1:400; Servicebio; Wuhan, China).

### 2.11 Transwell assay

Cells (2 × 10^4^) were added to the upper chamber, and assay medium (600 μL medium containing 30% FBS) was added to the lower chamber. After incubating at 37°C for 24–36 h, migrated or invaded cells were fixed and stained with crystal violet for 15 min. Images were obtained from three random fields (×100) in each well. Experiments were performed in triplicate.

### 2.12 Cell viability assay

Cell viability was determined using the CCK‐8 assay (#40203ES76, 10:100; Yeasen, Shanghai, China). Transfected cells were seeded into 96‐well plates (5 × 10^3^ cells/well) and incubated overnight. Then, CCK‐8 solution was added to each well every 24 h. After incubation for an additional 1 h at 37°C, each sample was measured at 450 nm by a microplate reader (Bio‐Rad, Hercules, CA, USA).

### 2.13 EdU assay

Cell proliferation was measured with an EdU assay kit (Ribo‐Bio, Guangzhou, China). Experiments were performed according to the manufacturer's instructions. Representative images were obtained using a Leica inverted fluorescence microscope.

### 2.14 Colony formation assay

After the indicated treatment, cells (1 × 10^3^/well) were added into six‐well plates and cultured for 2 weeks. Cells were fixed with 4% methanol and stained with 5% crystal violet, and the number of colonies per well was counted. Experiments were performed in triplicate.

### 2.15 Immunofluorescence staining

Cells were cultured in 8‐well confocal plates, fixed with 4% paraformaldehyde, permeabilized with 0.4% Triton X‐100, blocked with 5% bovine serum albumin, and incubated with primary antibody against PSMD9 (#67338‐1‐Ig, 1:400; ProteinTech, USA) at 4°C overnight. The primary antibody was detected with an Alexa Fluor 488‐conjugated goat anti‐mouse IgG antibody (#ab150080, 1:800; Abcam; USA), and cell nuclei were stained with DAPI (Sigma–Aldrich, St. Louis, USA), and the cytoskeleton was stained with TRITC (#40734ES75, 1:200; Yeasen, Shanghai, China). Images were obtained under fluorescence microscopy (Leica, Wetzlar, Germany).

### 2.16 Wound healing assay

LN229 and A172 cells, which were transfected and collected, were seeded into 6‐well plates. The monolayer was scratched with a pipette tip. Then, the cells were incubated in serum‐free medium for 0–72 h. Then, the final distance of the healed scratch wound was quantified using ImageJ software.

### 2.17 Western blotting

Protein lysates of cells were extracted by RIPA lysis buffer (#P0013B; Beyotime Biotechnology, Jiangsu, China). Equal volumes of protein samples were separated by 10% SDS/PAGE and electrotransferred to PVDF membranes. The immunoblots were incubated with the indicated antibodies overnight. The expression of the target protein was normalized to β‐Actin expression. The primary antibodies used were as follows: β‐Actin (#66009‐1‐Ig, 1:20,000; Protein Tech, USA), PSMD9 (#67338‐1‐Ig, 1:1000; Protein Tech, USA), cyclin B1 (#67686‐1‐Ig, 1:5000; Protein Tech, USA), CDK1 (#19532‐1‐AP, 1:1000; Protein Tech, USA), Vimentin (#ab92547, 1:5000; Abcam, USA) and N‐cadherin (#22018‐1‐AP, 1:2000; Protein Tech, USA).

### 2.18 Intracranial xenograft model

Luciferase‐stable LN229 cells (5 × 10^5^) in a total volume of 10 μL of PBS were implanted into the frontal lobes of nude mice (female; 5 weeks old; Shanghai SLAC Laboratory Animal Co., Ltd, Shanghai, China) using a stereotactic device (KDS310; KD Scientific, Holliston, MA, USA). The burr hole was positioned 1 mm anterior and 2 mm lateral from the anterior fontanel, and the injection depth was adjusted to 2.0 mm. For the panobinostat treatment groups, panobinostat was administered intraperitoneally at a dose of 10 mg/kg three times a week for 4 weeks. At days 7, 14 and 21 after implantation, tumor growth was examined using bioluminescence imaging (IVIS Spectral In Vivo Imaging System, PerkinElmer; Hopkinton, MA, USA). Body weight was measured every 3 days, and the mice were sacrificed when they began to show symptoms of continuous discomfort. The brains of mice were removed, fixed, embedded in paraffin and sectioned for IHC staining.

### 2.19 Statistical analysis

Statistical analyses were performed using GraphPad Prism version 8.0. All values are presented as the means ± standard deviation (SD). Data distributions were tested using a frequency distribution histogram. Differences were analyzed using Student's *t* test for two groups and one‐way ANOVA for multiple groups, while data that did not exhibit a normal distribution were analyzed via a nonparametric equivalent. Using Spearman and Pearson coefficients, relationships between variables were analyzed. For survival analyses, applying the Kaplan–Meier approach and log‐rank test, we acquired survival curves while assessing the statistical significance. A *p* value <0.05 was regarded as statistically significant.

## 3 RESULTS

### 3.1 The mRNA expression profiles of PSMD family genes in GBM

**FIGURE 1:**
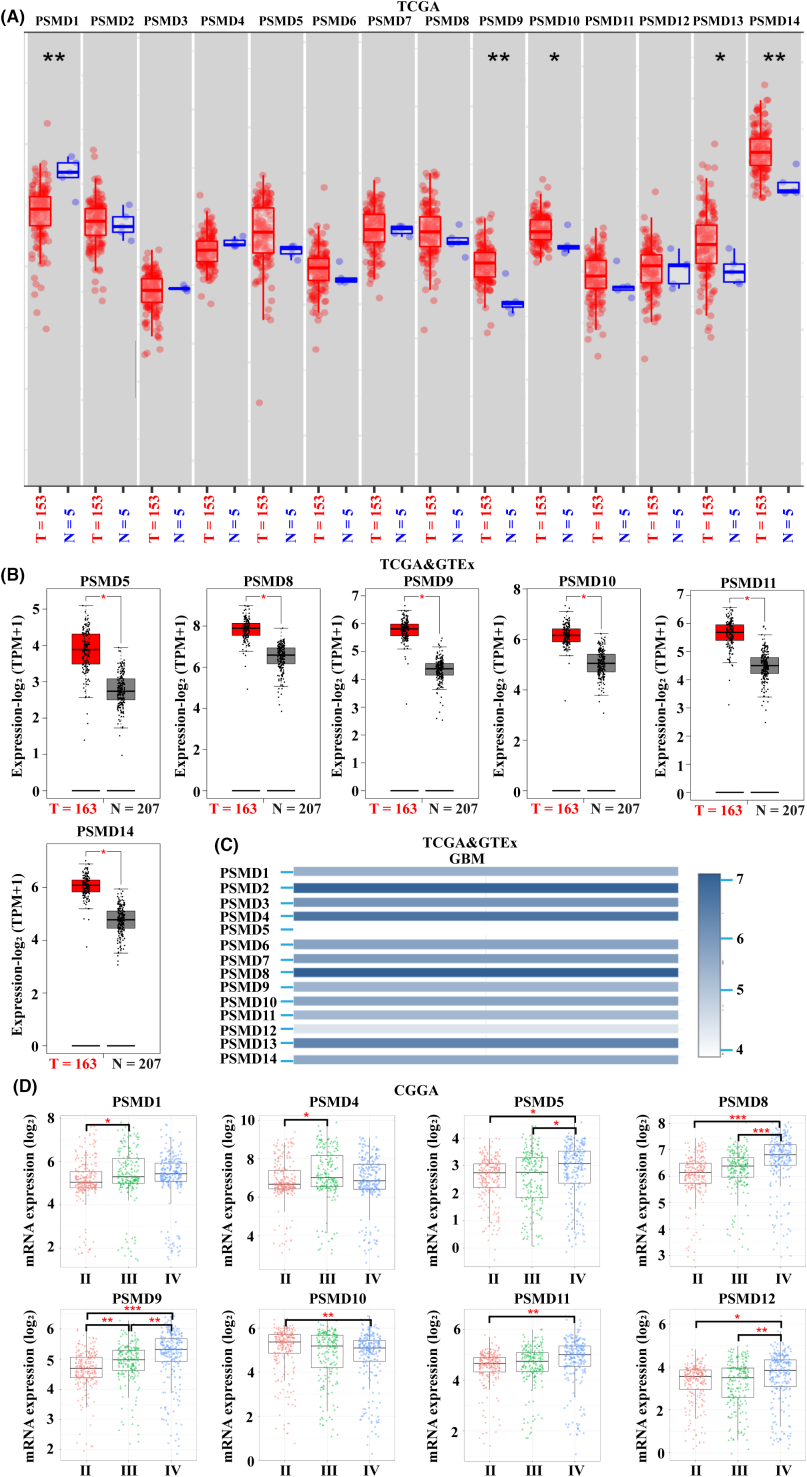
PSMD family mRNA expression levels in glioblastoma (GBM). (A) mRNA expression levels of 14 PSMD family members in GBM tissues and normal brain tissues from TIMER2. T: glioma tissues, N: normal tissues. (B) mRNA expression levels of six PSMD family members in GBM tissues and normal brain tissues from GEPIA2. T: GBM tissues, N: normal tissues. (C) The relative expression levels of 14 PSMD members in GBM from GEPIA2. (D) Relationships between the mRNA expression levels of eight PSMD family genes and the tumor grade of glioma from GlioVis. *p < 0.05, **p < 0.01, ***p < 0.001.

The graphical abstract image shows the study details for a comprehensive view. To investigate the differences in the expression of the PSMD family in GBM patients, the mRNA and protein expression levels of PSMD family genes were analyzed in different databases. According to the TIMER2 database (glioma tissues, *n* = 153; adjacent normal tissues, *n* = 5), PSMD9/10/13/14 mRNA expression levels were significantly increased in GBM, while PSMD1 mRNA expression levels were decreased in GBM tissues (Figure 1A). Then, according to GEPIA2 data (GBM tissues, *n* = 163; normal brain tissues, *n* = 207), we obtained similar results: the mRNA expression levels of PSMD5/8/9/10/11/14 were higher in GBM tissues than in normal brain tissues (Figure 1B; Figure S1A). We also compared the relative expression levels of PSMD members in GBM using the GEPIA2 database and found that the mRNA expression level of PSMD2 and PSMD8 was higher than that of the other PSMD family members, and the mRNA expression level of PSMD5 was the lowest among all 14 PSMD members (Figure 1C). The relationships between the mRNA expression of PSMD family members and glioma grade were investigated using the GlioVis database (glioma, *n* = 651). The mRNA expression of PSMD1/4/5/8/9/10/11/12 was significantly correlated with glioma grade, while the mRNA expression of PSMD2/3/6/7/13/14 was not correlated with glioma grade. Statistically, the mRNA expression of PSMD1/4/5/8/9/11/12 tended to increase as glioma grade increased, whereas the mRNA expression of PSMD10 tended to decrease with increasing glioma grade (Figure 1D; Figure S1B).

### 3.2 The protein expression of PSMD family members in GBM

**FIGURE 2:**
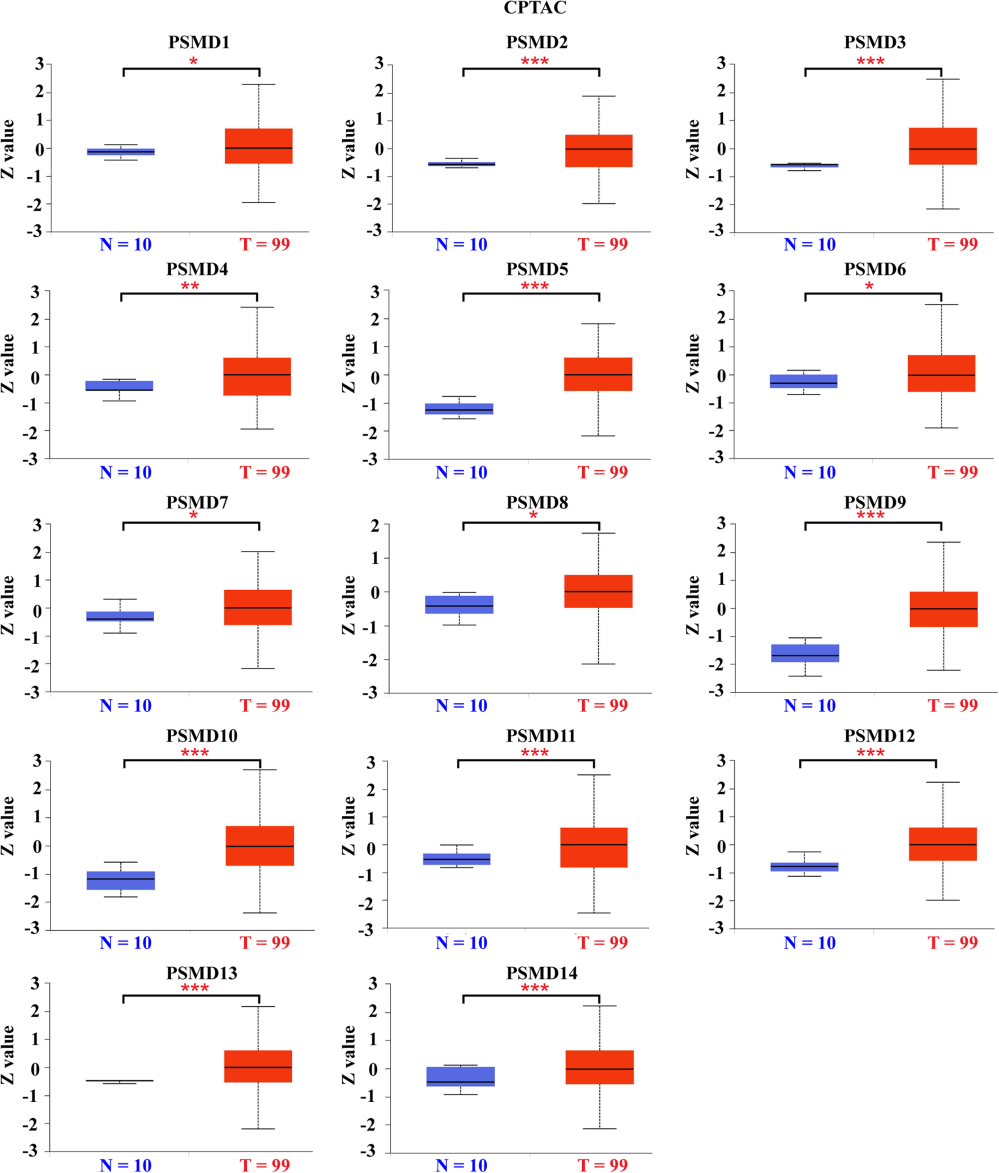
Protein expression levels of the PSMD family in glioblastoma (GBM). Analysis of protein expression levels of the PSMD family in GBM using the UALCAN database. T: GBM tissues, N: normal tissues. *p < 0.05, **p < 0.01, ***p < 0.001.

After studying the mRNA expression of PSMD family genes, we further explored their protein expression and localization in GBM cells. According to UALCAN analysis (normal brain samples, *n* = 10; GBM samples, *n* = 99), all PSMD family members had higher protein expression in GBM than in normal brain tissues (Figure 2). Similar to the results of the UALCAN database analysis, the IHC results showed that most of the PSMD members exhibited significantly higher expression levels in GBM tissues than in normal brain tissues; the exceptions were PSMD5/11, for which there was no obvious difference in expression levels. PSMD8/12/14 protein expression was not detected in normal brain tissues, which may result from its low expression in normal brain tissues (Figure S2A). As shown in Figure S2B, most of the PSMD members are localized in the nucleoplasm and cytosol toggle channels (PSMD3/4/5/9/14), and some are localized in microtubule toggle channels (PSMD12) or nucleoli and nuclear speckle toggle channels (PSMD13).

### 3.3 Prognostic value of PSMD family members in GBM patients

**FIGURE 3:**
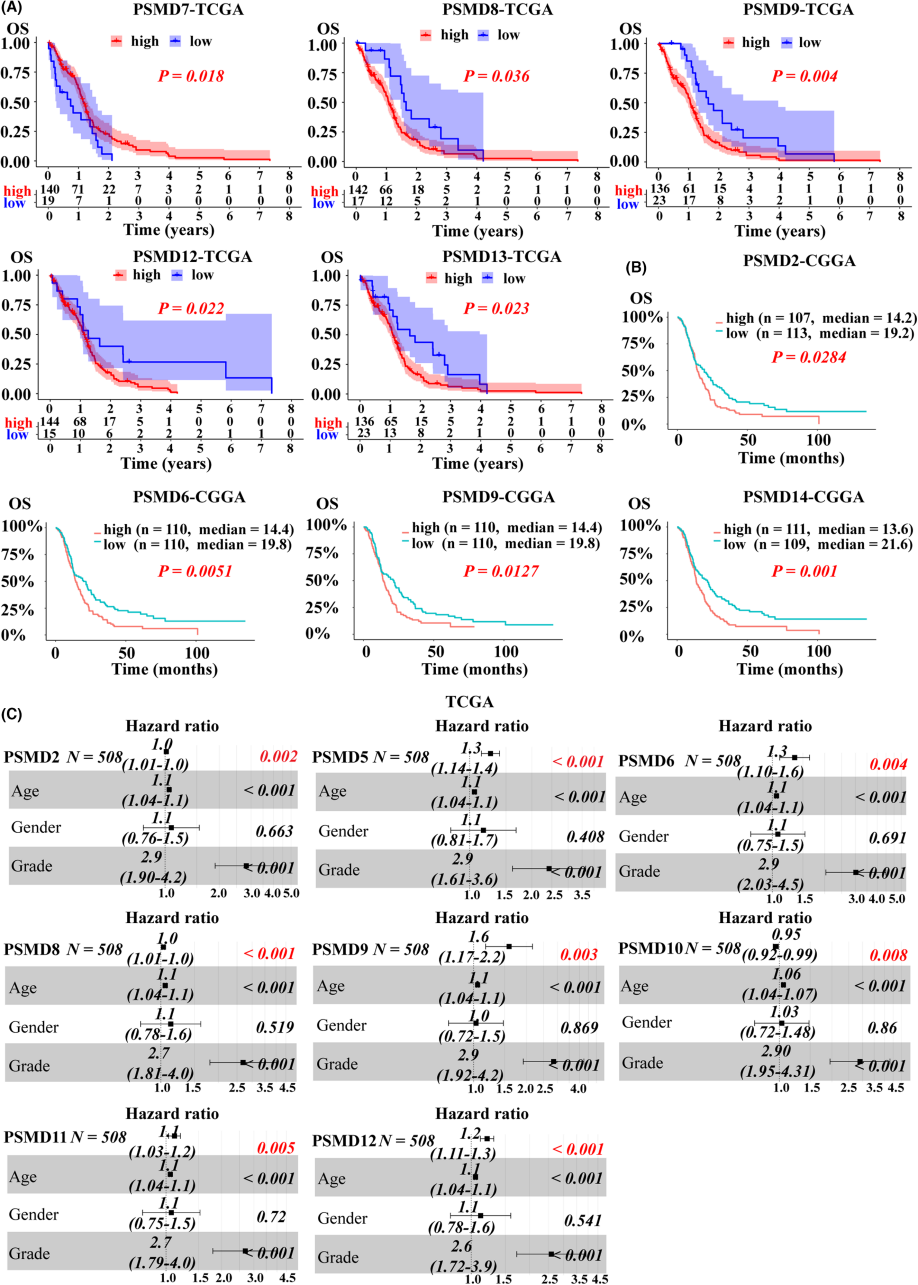
Prognostic performance of PSMD family genes expression in glioblastoma (GBM) patients. (A) Kaplan–Meier survival analyses of PSMD members from the TCGA database. (B) Kaplan–Meier survival analyses of PSMD members from the CGGA database. (C) Multivariate Cox analysis of the ability of PSMD expression and other clinicopathological variables to predict the OS of GBM patients. The results are presented as the hazard ratio (HR). The bar represents the 95% confidence interval (CI) of the HR values.

To gain insight into the prognostic value of PSMD in GBM patients, we analyzed the correlations between the expression of PSMD genes and patient survival time. By analyzing the data from the TCGA database (GBM samples, *n* = 701), we found that higher expression of PSMD8/9/12/13 was significantly correlated with shorter survival time, while overexpression of PSMD7 was associated with longer survival time (Figure 3A; Figure S3A). We also performed data analysis based on the CGGA (GBM samples, *n* = 220), and the results demonstrated that high expression of PSMD2/6/9/14 was obviously correlated with a poorer prognosis (Figure 3B; Figure S3B). Then, we analyzed the potential prognostic indicators, including patient age, patient sex, tumor grade, and PSMD expression level, by Cox regression analyses. The univariate analysis showed that patient age, tumor grade and PSMD2/5/6/8/9/10/11/12/14 expression levels were significantly associated with OS (*p* < 0.05) (Table 1). These associated risk factors were further included in multivariate Cox regression. The analysis showed that PSMD2/5/6/8/9/10/11/12 were independent prognostic factors, while PSMD14 was not (Figure 3C; Figure S3C).

**TABLE 1:** Validation of univariate analysis of variables related to OS in GBM from the TCGA database.

Characteristics	Univariate analysis	
	HR (95% CI)	*p* value
PSMD1	1.03904 (0.99814–1.08160)	0.06158
PSMD2	1.02005 (1.01136–1.02881)	<0.001*
PSMD3	1.02212 (0.98934–1.05599)	0.18834
PSMD4	0.98627 (0.96835–1.00453)	0.13957
PSMD5	1.34396 (1.21496–1.48666)	<0.001*
PSMD6	1.29807 (1.06522–1.58180)	<0.01*
PSMD7	1.04319 (0.99961–1.08868)	0.05213
PSMD8	1.04209 (1.02484–1.05963)	<0.001*
PSMD9	2.02217 (1.47703–2.76852)	<0.001*
PSMD10	0.94327 (0.90867–0.97919)	<0.01*
PSMD11	1.15361 (1.07597–1.23685)	<0.001*
PSMD12	1.29449 (1.19817–1.39855)	<0.001*
PSMD13	1.00086 (0.96604–1.03694)	0.96207
PSMD14	1.16765 (1.05303–1.29474)	<0.01*
Age (>40 vs. ≤40)	1.05803 (1.04311–1.07316)	<0.001*
Gender (Male vs. Female)	1.09707 (0.76896–1.56518)	0.60937
Grade (High vs. Low)	3.39671 (2.29640–5.02423)	<0.001*

*Note*: High: grade III, IV; Low: grade II.

Abbreviations: CI, Confidence interval; HR, Hazard ratio.

* *p* < 0.05, significant difference.

### 3.4 Genetic alteration analysis of PSMD family members

We analyzed the genetic alterations of PSMD members using DNA sequencing data from GBM patients that was obtained from the cBioPortal online database. Four GBM datasets were analyzed, and the results showed the frequency of gene alterations, including mutation, amplification and deep deletion. Amplification was the most frequently observed type of alteration (Figure S4A). The GSCA database was then utilized to investigate the roles of PSMD members in genetic alteration. The results suggested that the SNV frequencies of PSMD12, PSMD3, PSMD2, PSMD7, PSMD5, PSMD4, and PSMD11 were 17, 25, 25, 17, 17 and 17%, respectively (Figure S4B). In addition, the waterfall plot showed that missense mutations were the most common form of SNVs (Figure S4C).

### 3.5 Genetic methylation of PSMD family genes

Next, we performed a correlation analysis between mRNA expression and DNA methylation of PSMD to discover the effect of promoter region DNA methylation on PSMD expression with the GSCA platform. There was a negative correlation between the mRNA expression of PSMD2/3/5/7/13 and gene methylation levels in GBM (Figure S5A). The difference in survival between patients with high and low PSMD methylation was further analyzed. PSMD3/14 hypomethylation was associated with poorer OS (Figure S5B). The results of correlation analysis between PSMD2/3/5/7/13/14 methylation sites and gene expression from the TCGA dataset (GBM samples, *n* = 701) are shown in Figure S5C. As an example, the results revealed that cg00306249 and cg09604352 are the most likely methylation sites regulating PSMD2 expression, and cg09586646 is the most likely methylation site regulating PSMD5 expression (Figure S5C).

### 3.6 Correlation of PSMD expression with immune infiltration in GBM

A growing amount of evidence suggests that infiltrating immune cells and other stromal components in the tumor microenvironment are associated with the prognosis of GBM.^17^, ^18^ Then, the TIMER2 database was used to evaluate whether the expression of PSMD in GBM was correlated with immune cell infiltration. Interestingly, we found a significant correlation between the expression of PSMD and immune cell infiltration. For example, the PSMD2 expression level had a significant positive correlation with the level of dendritic cells (*r* = 0.138, *p* = 4.27e‐03) and a significant negative correlation with the level of infiltrating B cells (*r* = −0.138, *p* = 4.58e‐03; Figure S6).

### 3.7 The effect of PSMD family genes on tumorigenesis and progression

**FIGURE 4:**
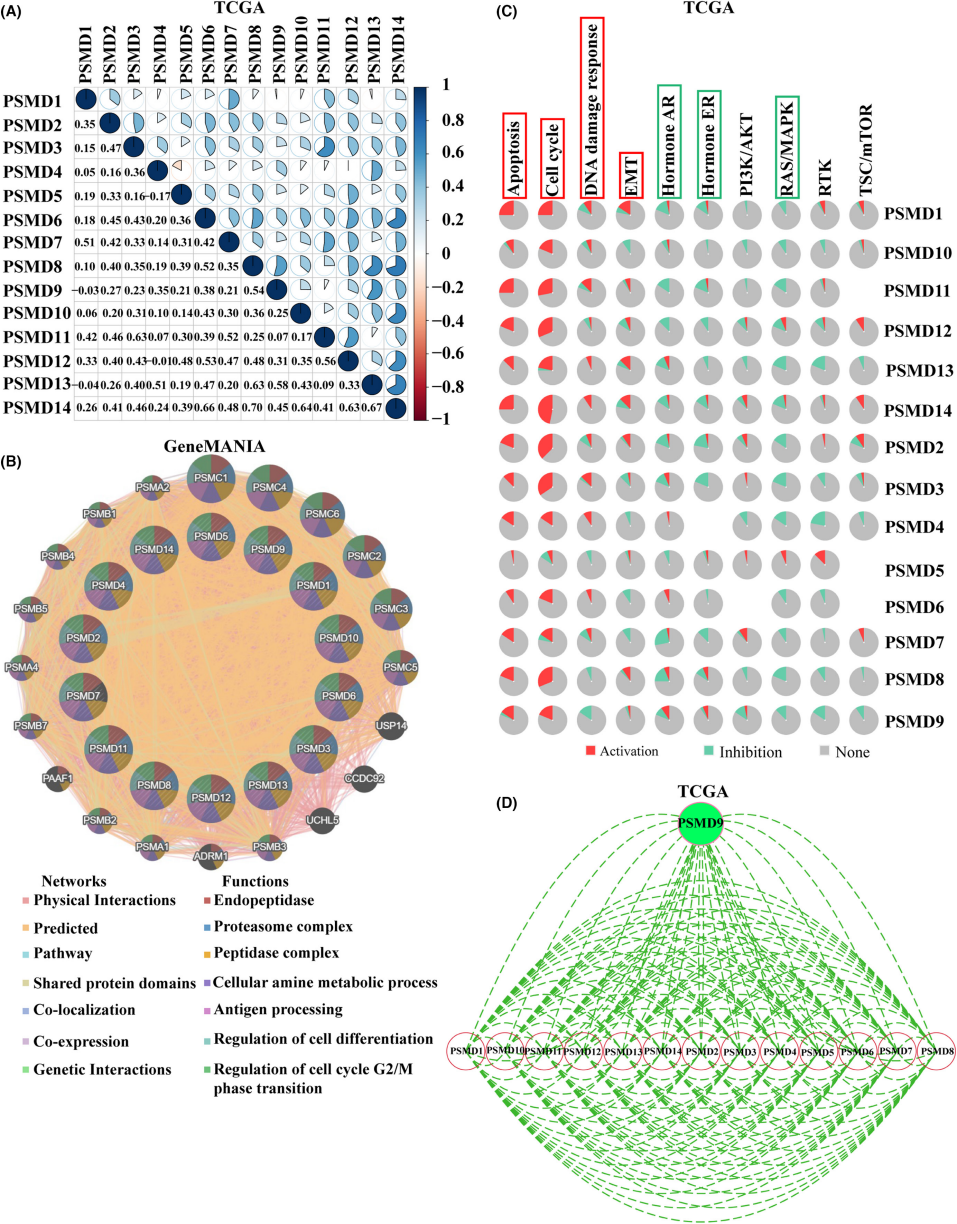
The effect of PSMD family members on tumorigenesis and progression. (A) The coexpression of PSMD members based on the TCGA database. (B) Gene–gene interaction network among PSMD members in GeneMANIA. (C) The roles of PSMD family genes in common cancer‐related pathways from GSCA. (D) The protein–protein interaction network of PSMD family was assessed by STRING. EMT, epithelial‐mesenchymal transition.

To understand the gene family coexpression relationships, we performed expression correlation analysis with the TCGA dataset. The results showed that most PSMD members were positively correlated with each other (Figure 4A). Then, we analyzed the relationships between PSMD members at the gene level using GeneMANIA. The results showed that most PSMD members shared protein domains and had physical interactions and coexpression relationships. The GeneMANIA results also showed that the functions of differentially expressed PSMD members and their related molecules were mainly associated with the proteasome complex, cellular amine metabolic process, peptidase complex and regulation of the cell cycle G2/M phase transition (Figure 4B). In addition, we analyzed the role of PSMD in the activities of several well‐known cancer‐related pathways. The results from GSCA showed that PSMD members are mainly involved in the apoptosis, cell cycle, DNA damage response, EMT, hormone AR, hormone ER and RAS/MAPK pathways (Figure 4C). Subsequently, the interaction network of PSMD expression at the protein level was constructed. The STRING protein–protein interaction network analysis revealed that there are complex interactions among PSMD family members (Figure 4D).

### 3.8 GSEA investigation of PSMD‐related pathways

Based on the RNA sequencing data from the TCGA database (GBM samples, *n* = 701), GSEA was further performed using the “ggplot2” package to characterize the biological functions of PSMD members at different expression levels. Since PSMD2/5/6/8/9/10/11/12 were validated as independent prognostic factors in Figure 3C, they were used as the focus of GSEA. The results showed that gene sets from proliferation‐, migration‐ and invasion‐related pathways, including the cell cycle, P53, proteasome, ubiquitin‐mediated proteolysis, and pyrimidine metabolism pathways, were preferentially enriched in the high‐PSMD GBM samples (Figure S7).

### 3.9 Prognostic prediction model of PSMD9 in GBM

**FIGURE 5:**
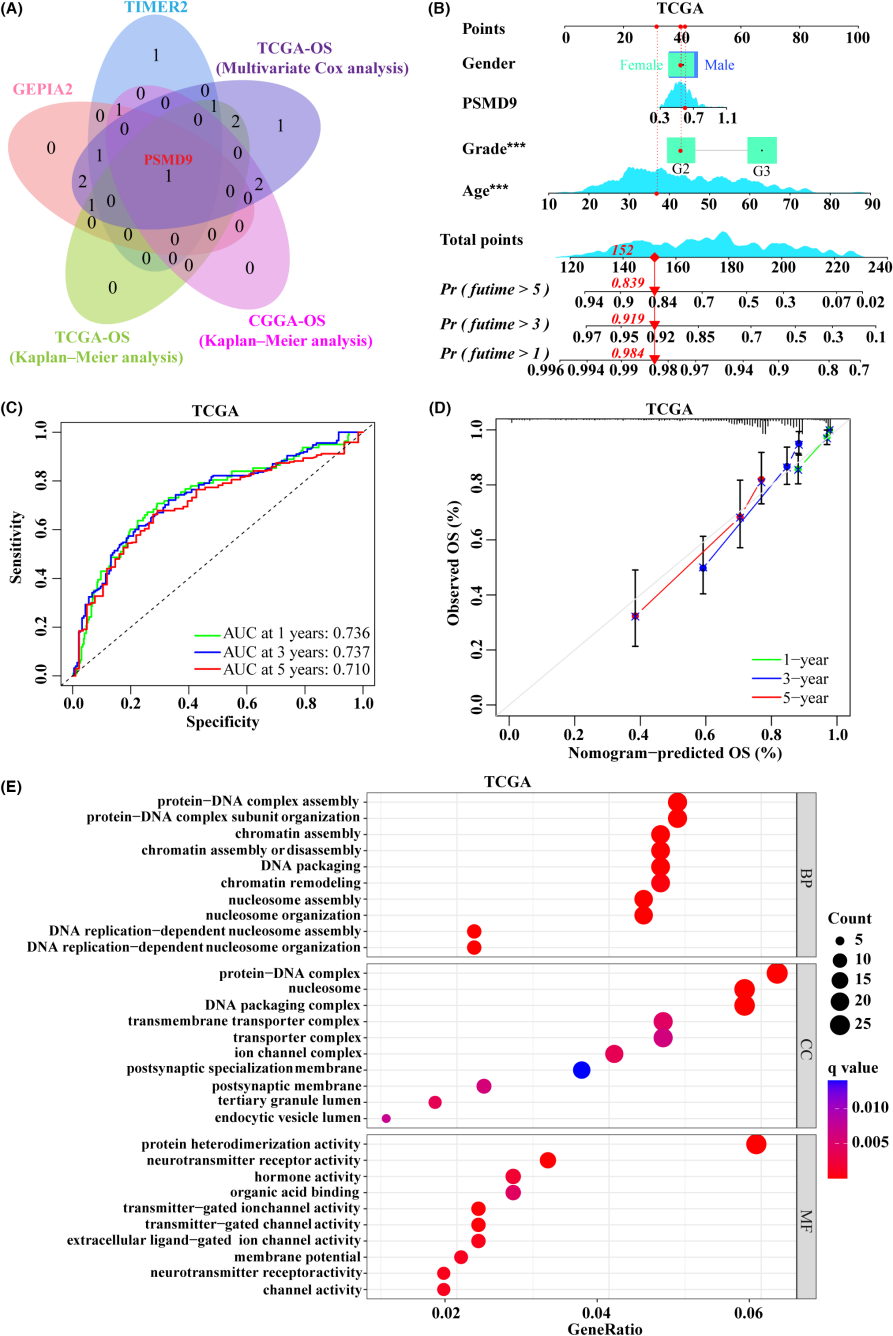
Construction and validation of a prognostic nomogram for PSMD9. (A) Identification of relatively important genes in the PSMD family by Venn diagram. (B) A nomogram for OS prediction of glioblastoma patients in TCGA, with gender, age, grade, and the expression level of PSMD9 applied as parameters. (C) Time‐dependent ROC curves and AUC values for 1‐, 3‐ and 5‐year OS prediction. (D) Calibration curves for 1‐, 3‐ and 5‐year OS prediction. (E) GO functional enrichment analysis of PSMD9, including BP, CC and MF categories.

A Venn diagram was used to show the relationships between the expression results of PSMD from the TIMER2 and GEPIA2 databases and the survival analysis results from the TCGA and CGGA. Based on the results, we found that the mRNA expression of PSMD9 was higher in GBM tissues than in normal brain tissues in both TIMER2 and GEPIA2, and it had prognostic value in both TCGA and CGGA (Figure 5A). Then, a nomogram was constructed using gender, age, grade, and PSMD9 expression level as indicators to predict 1‐, 3‐, and 5‐year OS in GBM patients based on TCGA data (GBM samples, *n* = 701) (Figure 5B). Subsequently, we developed time‐dependent ROC curves and calibration plots predicting the probability of 1‐, 3‐ and 5‐year OS rates. The areas under the curves (AUCs) for 1‐, 3‐, and 5‐year survival were 0.736, 0.737, and 0.710, respectively (Figure 5C). The calibration curve showed that the nomogram performed well in predicting the 1‐, 3‐, and 5‐year clinical outcomes (Figure 5D). We performed gene ontology (GO) functional annotation analysis of PSMD9, including biological process (BP), cellular component (CC) and molecular function (MF) analyses, and the results showed that PSMD9 was primarily associated with protein–DNA complex assembly or organization in the BP category. In addition, PSMD9 was mainly associated with the protein–DNA complex and nucleosome in the CC category. PSMD9 was associated with protein heterodimerization activity or neurotransmitter receptor activity in the MF category (Figure 5E). Taken together, the above data illustrated that PSMD9 is a crucial factor in GBM and might serve as a useful biomarker for the prediction of OS among GBM patients.

### 3.10 PSMD9 loss‐of‐function attenuated GBM cell proliferation in vitro

**FIGURE 6:**
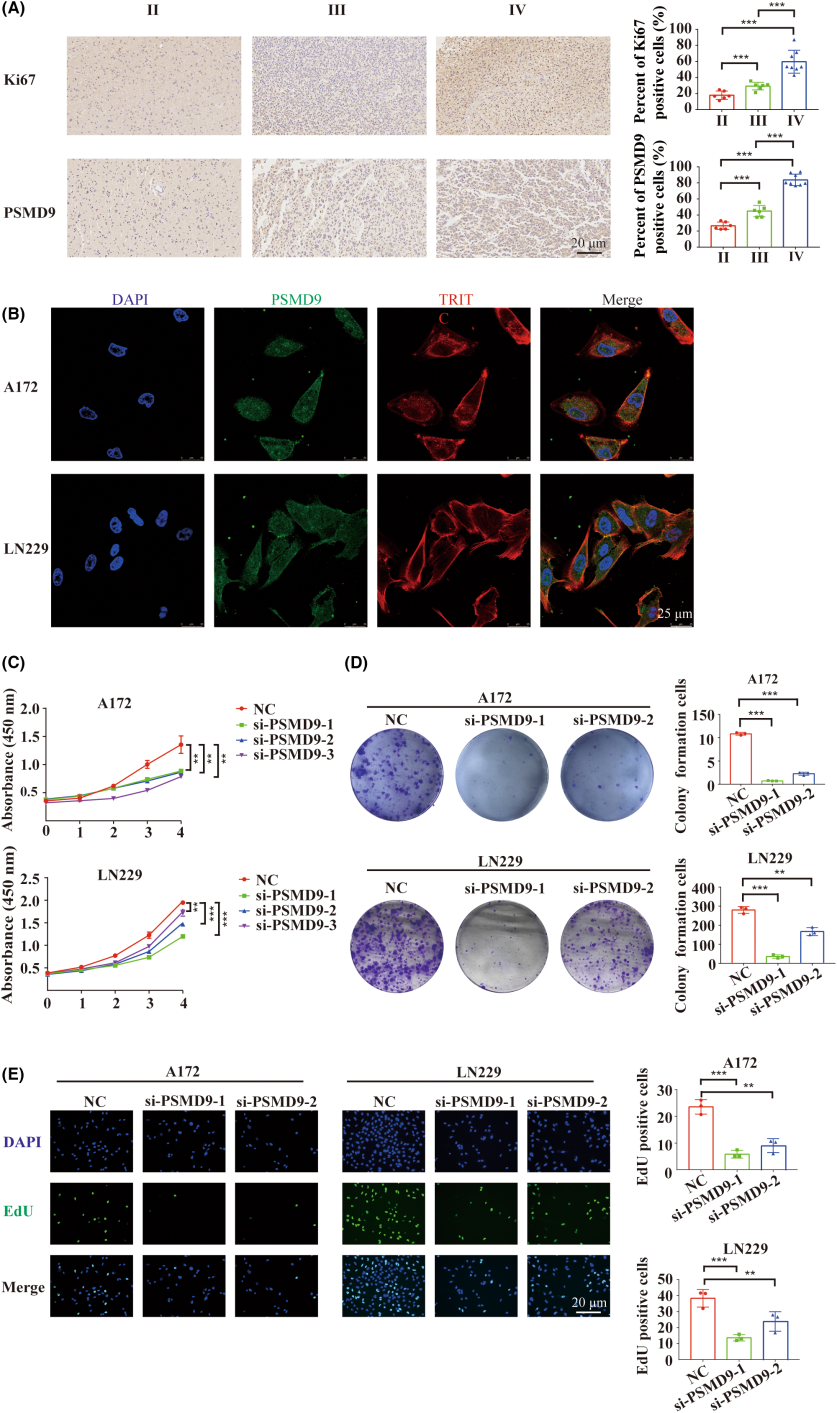
PSMD9 was upregulated in glioblastoma, and loss of PSMD9 function attenuated cell proliferation. (A) The expression levels of PSMD9 and Ki67 in glioma tissues of different grades were determined by IHC staining. (B) Immunofluorescence images demonstrated the protein localization of PSMD9. (C) CCK‐8 assays were used to assess the viability of LN229 and A172 cells transfected with PSMD9 siRNAs and control siRNAs. (D) Colony formation assays were used to assess the viability of LN229 and A172 cells transfected with PSMD9 siRNAs and control siRNAs. (E) EdU assays for LN229 and A172 cells transfected with PSMD9 siRNAs or control siRNAs. **p < 0.01, ***p < 0.001.

Initially, differential expression analysis of PSMD9 was performed on glioma tissues of different grades, which revealed that increased protein expression of PSMD9 is associated with higher glioma grade (Figure 6A). The immunofluorescence results showed that PSMD9 in A172 and LN229 cells was mainly localized in the cytoplasm, which was generally consistent with the results of GBM U251 cells in Figure S2B (Figure 6B). To examine the function of PSMD9 in GBM development, we knocked down the expression of PSMD9 in GBM cells in culture with small interfering RNAs (siRNAs). Cell growth was significantly suppressed in cells expressing the PSMD9 siRNAs compared with those expressing negative controls (NC) according to three different assays (CCK‐8, colony formation, and EdU assays) (Figure 6C–E).

### 3.11 PSMD9 loss‐of‐function caused G2/M cell cycle arrest and attenuated cell invasion and migration

**FIGURE 7:**
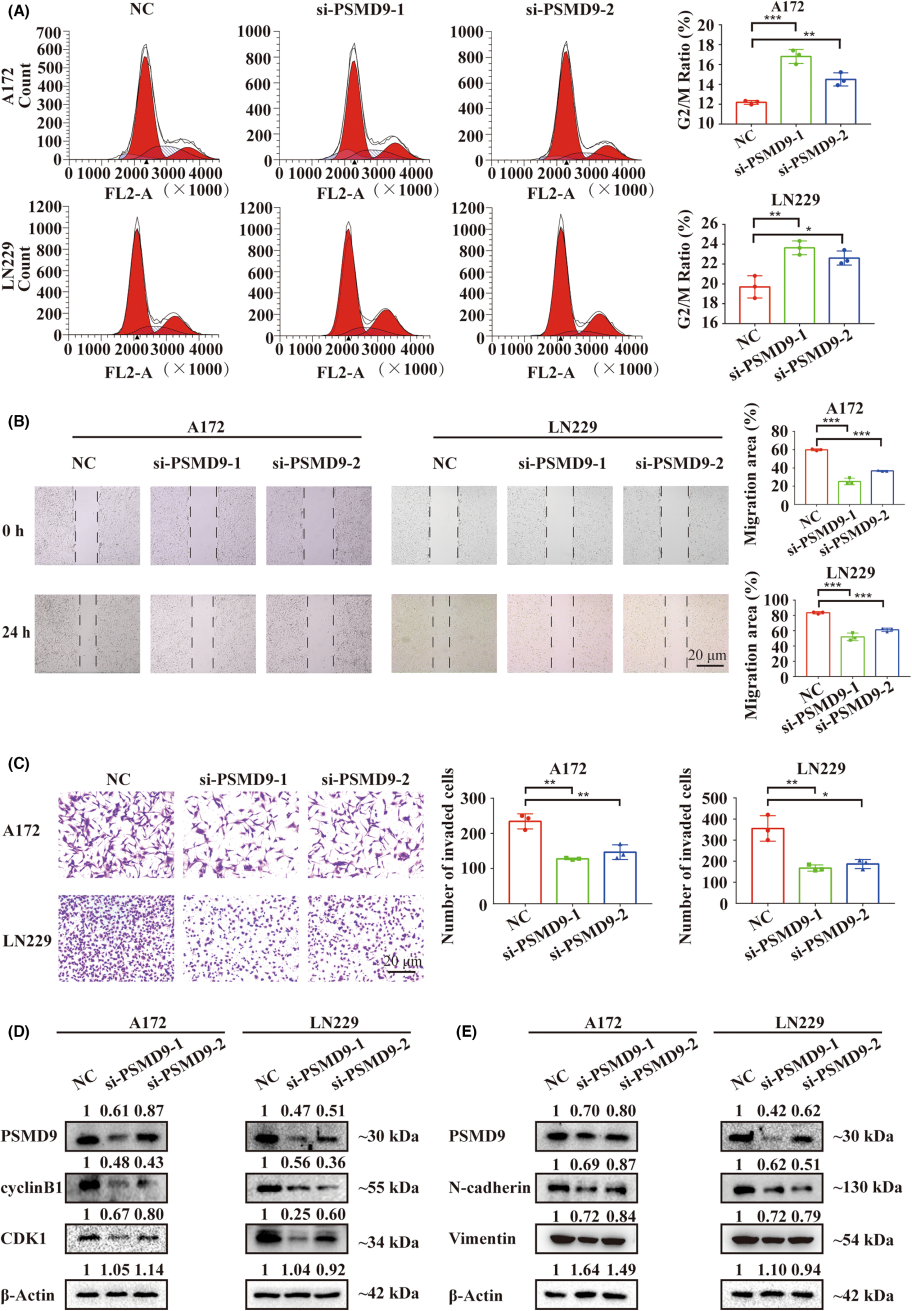
Knockdown of PSMD9 caused G2/M cell cycle arrest and attenuated glioblastoma cell invasion and migration. (A) Cell cycle distribution of LN229 and A172 cells determined with propidium iodide staining by flow cytometry analysis. Data points represent the percentage of cells in G2/M in LN229 and A172 at 48 h after siRNA transfection. (B) Wound healing assays for LN229 and A172 cells transfected with PSMD9 siRNAs or control siRNAs. (C) Transwell assays for LN229 and A172 cells transfected with PSMD9 siRNAs or control siRNAs. (D‐E) Western blot showing the expression levels of PSMD9, cyclin B1, CDK1, N‐cadherin, Vimentin and β‐Actin (protein loading control) in LN229 and A172 cells transfected with PSMD9 siRNAs or control siRNAs for 48 h. *p < 0.05, **p < 0.01, and ***p < 0.001.

Based on the functional enrichment analysis results of Figure 4 and Figure S7, we continued to investigate the effect of PSMD9 on the cell cycle, invasion and migration. Examination of the cell cycle showed that PSMD9 siRNA transfection led to an increase in cells in G2/M phase (Figure 7A). Furthermore, loss of PSMD9 suppressed the invasion and migration of LN229 and A172 cells, as assessed by wound healing assays and transwell assays (Figure 7B,C). The Western blotting results indicated that knockdown of PSMD9 led to a decrease in the expression of the cell cycle‐related proteins cyclinB1 and CDK1 as well as that of the cell invasion‐ and migration‐related proteins N‐cadherin and Vimentin in siRNA‐transfected LN229 and A172 cells (Figure 7D,E). Collectively, these data suggested that knockdown of PSMD9 inhibits the proliferation, migration and invasion of GBM cells in vitro.

### 3.12 Potential therapeutic value of PSMD9 expression

**FIGURE 8:**
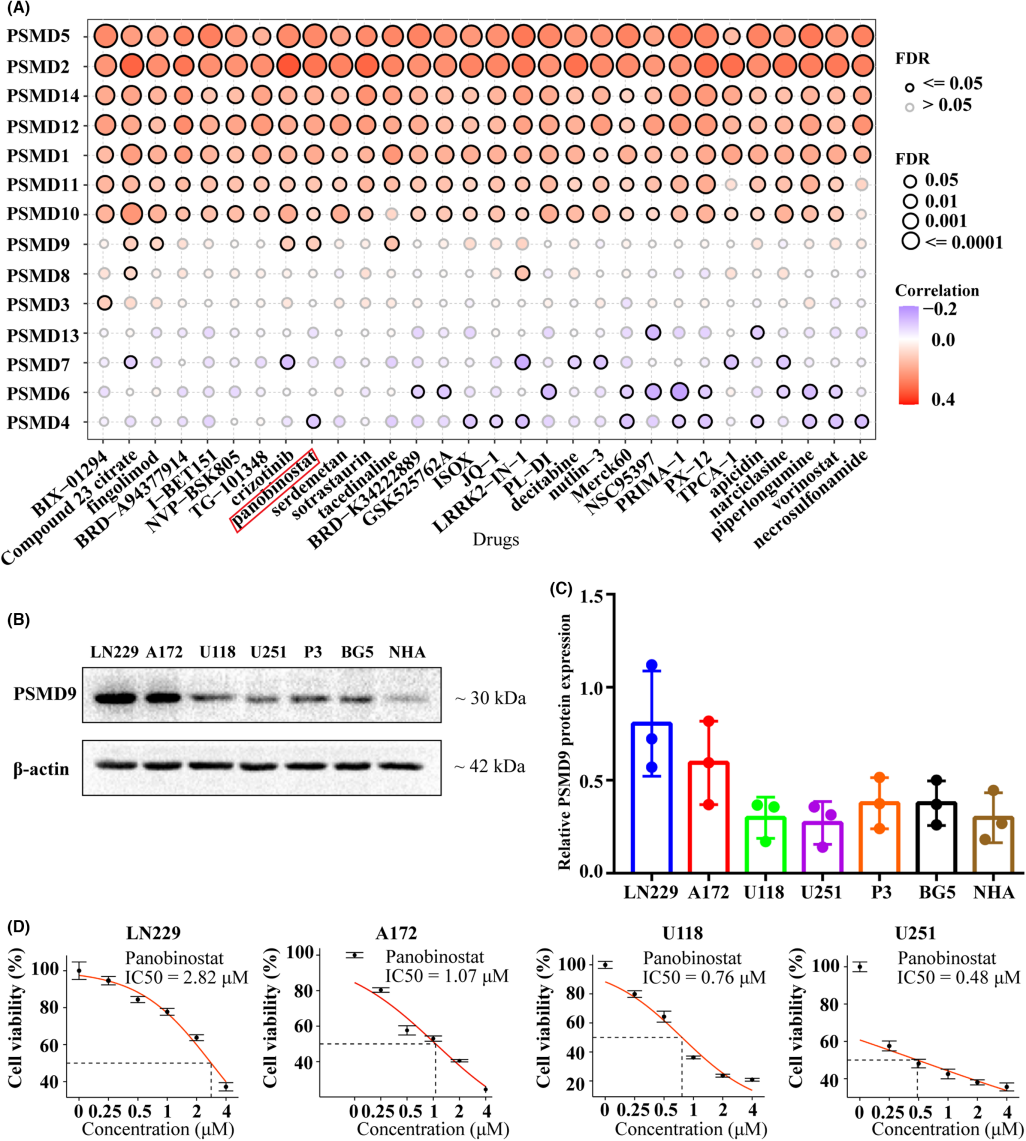
The relationship between PSMD expression and drug sensitivity. (A) Dot plot visualizing the relationship between PSMD expression and drug sensitivity from GSCA. A positive correlation (red) means that high expression of a gene indicates resistance to the drug, and vice versa. (B and C) Western blot analysis was performed to assess the expression levels of PSMD9 in NHAs and six GBM cell lines. (D) Representative panobinostat dose response curves of LN229, A172, U118 and U251 cells assessed by CCK‐8 assays at 24 h posttreatment.

We assessed the correlations between PSMD expression and the response to drugs in GBM cell lines. We identified the top 30 drugs for which PSMD expression and drug sensitivity were obviously correlated in the GSCA database (Figure 8A). There was a similar correlation between PSMD1/2/5/9/10/11/12/14 expression and drug sensitivity, and PSMD4/6/7/13 had similarities to each other. For example, the expression of PSMD1/2/5/9/10/11/12/14 was positively correlated with the sensitivity to panobinostat; the expression of PSMD4/7 was negatively related to the sensitivity to LRRK2‐IN‐1; and the expression of PSMD6/13 was negatively related to the sensitivity to NSC95397. In addition, U251 cells had the lowest PSMD9 expression, while LN229 cells had the highest PSMD9 expression among common GBM cell lines (Figure 8B,C). CCK‐8 assays further demonstrated that LN229 cells with high PSMD9 expression were strikingly more resistant to panobinostat than those with low PSMD9 expression (Figure 8D). These findings suggested that the expression of PSMD9 is associated with drug sensitivity and can therefore be used as a potential biomarker for designing effective treatment options.

### 3.13 PSMD9 overexpression reverses panobinostat‐induced suppression of GBM cell proliferation, invasion and migration in vitro

**FIGURE 9:**
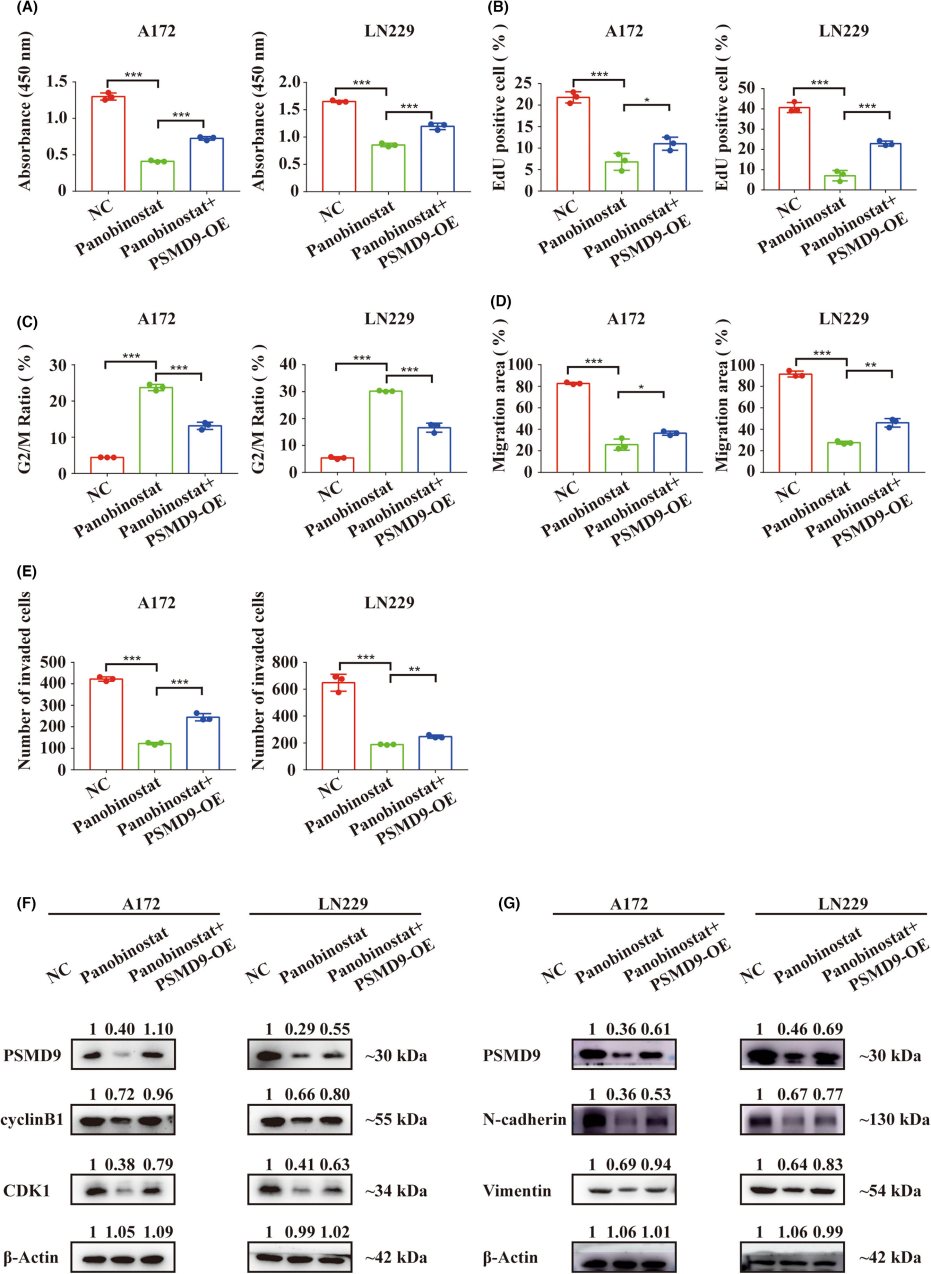
Overexpression of PSMD9 reverses the anti‐glioblastoma effect of panobinostat in vitro. (A) Cell viability of LN229 and A172 cells under the conditions indicated, as determined with CCK‐8 assays. (B) Analysis of EdU assays for LN229 and A172 cells, showing that ectopic expression of PSMD9 rescues the inhibitory effect of panobinostat on the proliferation of glioblastoma cells. (C) Percentage of LN229 and A172 cells in the G2/M phase under the conditions indicated. (D) Analysis of wound healing assays for LN229 and A172 cells under the conditions indicated. (E) Results of transwell assays for LN229 and A172 cells under the conditions indicated. (F‐G) Western blot to determine the levels of protein markers of PSMD9, cell cycle, invasion and migration in LN229 and A172 cells under the conditions indicated. Data are shown as the mean ± SD, and the differences between groups were analyzed with Student's *t* test. *p < 0.05, **p < 0.01, ***p < 0.001.

To determine whether increased PSMD9 expression interfered with panobinostat‐induced growth inhibition in GBM cells, we transiently transfected PSMD9 overexpression (OE) constructs into GBM cells. Overexpression of PSMD9 reversed the decrease in cell viability and proliferation caused by panobinostat as assessed by CCK‐8 and EdU assays for LN229 and A172 cells (Figure 9A,B; Figure S8A). Panobinostat‐induced G2/M cell cycle arrest was rescued in treated cells with ectopic expression of PSMD9 relative to treated controls (Figure 9C; Figure S8B). Overexpression of PSMD9 in cells also promoted the invasion and migration of GBM cells in the presence of panobinostat (Figure 9D,E; Figure S8C,D). Moreover, overexpression of PSMD9 partially reversed the changes in G2/M cell cycle arrest and invasion/migration markers caused by panobinostat (Figure 9F,G).

### 3.14 Downregulation of PSMD9 inhibits GBM growth, and overexpression of PSMD9 reverses panobinostat‐induced inhibition of GBM in vivo

**FIGURE 10:**
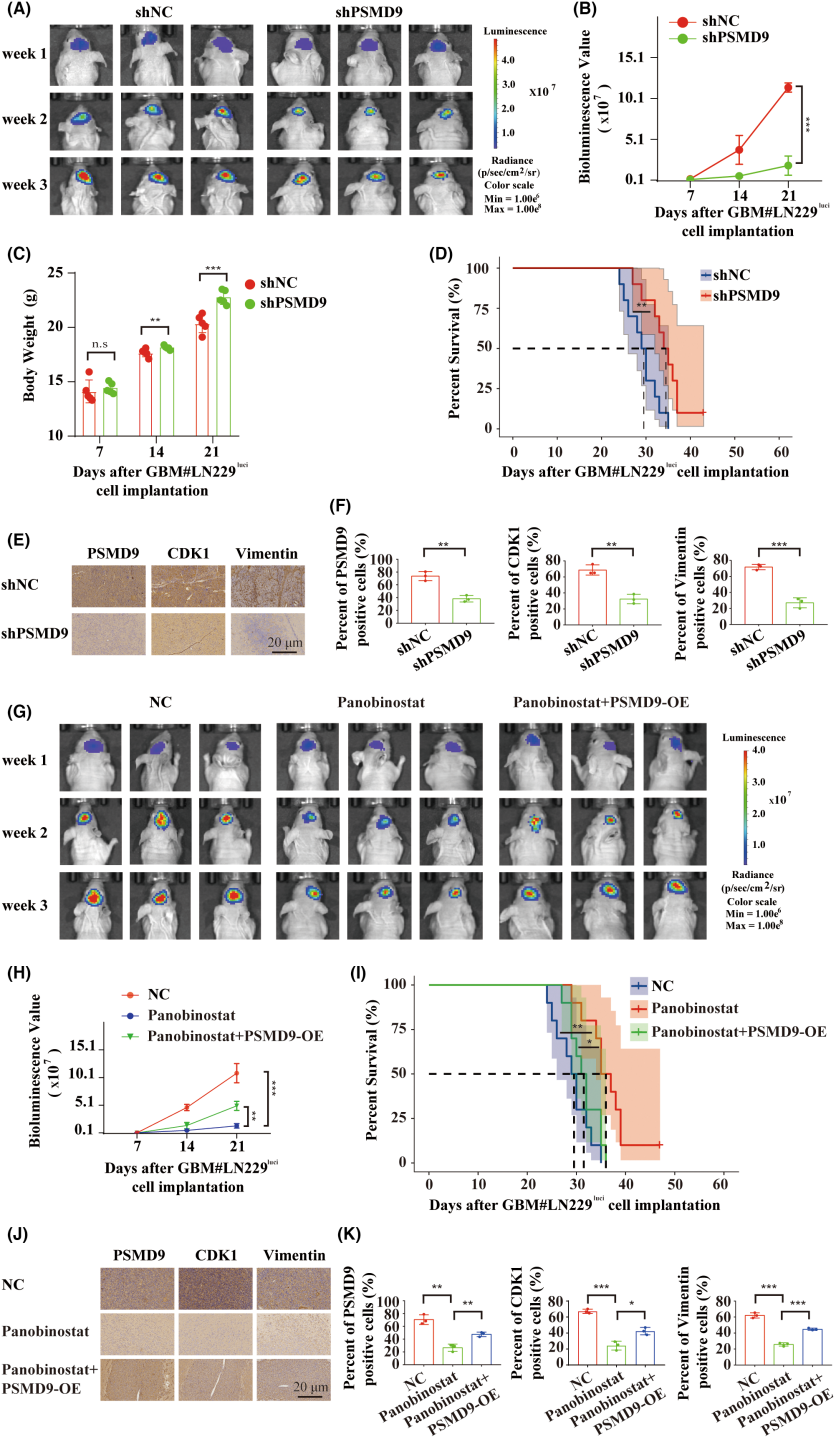
PSMD9 promotes glioblastoma progression and reverses the anti‐glioblastoma effect of panobinostat in vivo. (A and B) In vivo bioluminescence imaging and quantification of xenografts derived from lentivirus‐infected LN229 shNC and shPSMD9 cells at the indicated time points. (C and D) Weight curve and Kaplan–Meier curves of survival time for nude mice implanted with LN229 shNC and shPSMD9 cells at the indicated time points. (E and F) Representative images of IHC of PSMD9, CDK1 and Vimentin in xenograft sections (scale bar, 20 μm). (G and H) Bioluminescence imaging and quantification of xenografts derived from the LN229 PSMD9‐NC group, PSMD9‐NC panobinostat treatment group and lentivirus‐infected PSMD9‐OE + panobinostat treatment group at the indicated time points. (I) Kaplan–Meier analysis of survival time for nude mice at the indicated time points. (J and K) Representative images of IHC of PSMD9, CDK1 and Vimentin in xenograft sections (scale bar, 20 μm). Comparisons between two independent samples and among multiple samples were performed using two‐tailed *t* tests and one‐way ANOVA, respectively. Error bars indicate at least three independent experiments, and data are shown as the mean ± SD. n.s = no significance. *p < 0.05, **p < 0.01 and ***p < 0.001.

To determine the effect of PSMD9 on cell growth in vivo, we implanted luciferase‐expressing LN229‐shNC and LN229‐shPSMD9 cells into the brains of nude mice (*n* = 10 per group). Tumor size was significantly reduced for LN229‐shPSMD9 xenografts relative to controls on the 14th and 21st day after implantation (Figure 10A,B), while the body weight of the mice was increased to some extent relative to that of control animals (Figure 10C). Kaplan–Meier analysis revealed that the survival of mice with LN229‐shPSMD9 tumors was prolonged relative to that of controls (Figure 10D). IHC in sections from mice demonstrated that the protein expression of PSMD9, CDK1 and Vimentin was significantly reduced in the LN229‐shPSMD9 group (Figure 10E,F). Overall, the in vivo results further demonstrated a role for PSMD9 as an oncogene in human GBM.

To further investigate the effect of PSMD9 expression on the role of panobinostat in vivo, we constructed xenograft mouse models through orthotopic implantation of LN229‐PSMD9‐OE cells. The results showed that OE of PSMD9 partially reversed the inhibitory effect of panobinostat on GBM proliferation and attenuated the extension of the survival period of tumor‐bearing mice induced by panobinostat (Figure 10G–I). The IHC results of mouse sections showed that OE of PSMD9 significantly rescued the decrease in PSMD9, CDK1 and Vimentin protein expression caused by panobinostat (Figure 10J,K). Together, these in vivo results demonstrated the oncogenic function of PSMD9 and the therapeutic effect of panobinostat on GBM‐targeted PSMD9.

## 4 DISCUSSION

Despite the tremendous efforts made in recent years to explore the molecular biology of GBM and to continuously improve the treatment, most therapies have little effect in treating GBM.^19^, ^20^ Therefore, it is essential to investigate new diagnostic tools and new biomarkers that will allow us to study effective therapeutic measures. The UPS is the major protein proteolytic mechanism in human cells and plays a novel role in regulating cell survival and apoptosis.^21^, ^22^, ^23^ In recent years, the rapid development of microarray and high‐throughput sequencing technology has provided a convenient and comprehensive platform for more accurate elucidation of tumor pathogenesis mechanisms, surveillance of tumor progression, and prognosis evaluation.^24^, ^25^ Therefore, integrative bioinformatics analysis of the PSMD family was performed to explore the expression level, prognostic value and potential functions of PSMD members in GBM. To our knowledge, this is the first original study that concentrates on the value of the PSMD family in GBM. This may be useful for further development of PSMD‐based GBM diagnosis and treatment strategies.

Most genes of the PSMD family are upregulated in many types of cancer, such as lung adenocarcinoma, breast cancer, gastric cancer and hepatocellular carcinoma, and play an important role in their oncogenesis and progression.^26^, ^27^, ^28^, ^29^, ^30^, ^31^ In this study, we assessed the differential mRNA expression patterns of the PSMD family in GBM and adjacent normal brain tissues with the TIMER2 and GEPIA2 databases. The results showed that the mRNA expression of PSMD5/8/9/10/11/13/14 was higher in GBM tissues than in normal brain tissues. Kaplan–Meier analysis showed that high PSMD2/6/8/9/12/13/14 mRNA expression levels were strongly associated with poor OS in GBM. Several studies have suggested that high PSMD7 levels predict poor OS in LUAD, breast cancer and neck squamous cell carcinoma patients.^29^, ^32^, ^33^ However, PSMD7 expression has the opposite implication in GBM. Therefore, we will further focus on the expression of PSMD7 and its prognostic value in GBM. Moreover, based on our coexpression and pathway analysis, we found that the PSMD family is involved in pathways related to cell proliferation, invasion and migration, such as the cell cycle, apoptosis, cell metabolism, and EMT pathways. This is consistent with previous reports on the roles of PSMD members in some cancers.^29^, ^30^, ^31^

In particular, we found that PSMD9 plays a core role according to the mRNA expression analysis of TIMER2 and GEPIA2 databases and survival analysis of the TCGA and CGGA. The differential expression of PSMD9 in GBM cell lines and glioma tissues of different grades was confirmed using western blotting and IHC. We further explored the mechanisms underlying the prognostic value of PSMD9 in GBM using a series of in vitro and in vivo experiments. Consistent with functional enrichment analysis, the results showed that knockdown of PSMD9 inhibited the proliferation, invasion and migration of GBM cells and caused G2/M cell cycle arrest. Because there are no reports on the roles of PSMD9 in GBM, we intend to further investigate the specific mechanisms by which it functions in GBM.

Moreover, we evaluated the potential therapeutic effects of PSMD9. In GBM, differences in the expression of PSMD members affect sensitivity to chemotherapeutic agents. High expression of PSMD1/2/5/9/10/11/12/14 causes GBM cells to be resistant to drug therapy, while high expression of PSMD4/6/7/13 leads to sensitivity of GBM cells to certain chemotherapeutic agents. As shown in Figure 8, the higher the expression of PSMD9 was in a given GBM cell line, the less sensitive it was to the chemotherapeutic agent panobinostat. The results of in vivo and in vitro experiments further demonstrated that overexpression of PSMD9 reverses the inhibitory effects of panobinostat on GBM proliferation, invasion and migration. Therefore, the expression of PSMD9 may be considered a clinical target and a biomarker for predicting chemotherapy outcomes.

## 5 CONCLUSIONS

In conclusion, our study comprehensively explored the expression, prognostic value, methylation characteristics, genetic alterations, and potential functions of PSMD family members and the correlation between PSMD expression and immune infiltration and drug sensitivity. PSMD9 can serve as a diagnostic and prognostic marker for personalizing GBM therapy; panobinostat inhibits GBM progression by targeting PSMD9 and provides a valuable potential treatment for GBM.

## AUTHOR CONTRIBUTIONS

Anjing Chen and Bin Huang designed the study and wrote the original draft. Yaquan Li and Xuemeng Liu performed bioinformatics analysis and conducted experiments. Feihu Zhao, Zhimin Zhao, Xingang Li and Jian Wang collected and analyzed the data. All authors edited and revised the manuscript and agreed to be accountable for the content of the study.

## CONFLICT OF INTEREST STATEMENT

The authors declare no conflicts of interest.

## Supporting information


Figures S1–S8

Appendix S1

## Data Availability

All the datasets analyzed for this study can be accessed from the corresponding authors on reasonable request.
