# Supplementary material for: Comprehensive analysis of PSMD family members and validation of PSMD9 as a potential therapeutic target in human glioblastoma
Source: CNS Neurosci Ther. 2023 Jul 23;30(2):e14366. doi: 10.1111/cns.14366 (PMC10848081; doi:10.1111/cns.14366)
Supplement: Supplementary file 1 — Figures S1–S8 [file CNS-30-e14366-s001.docx]

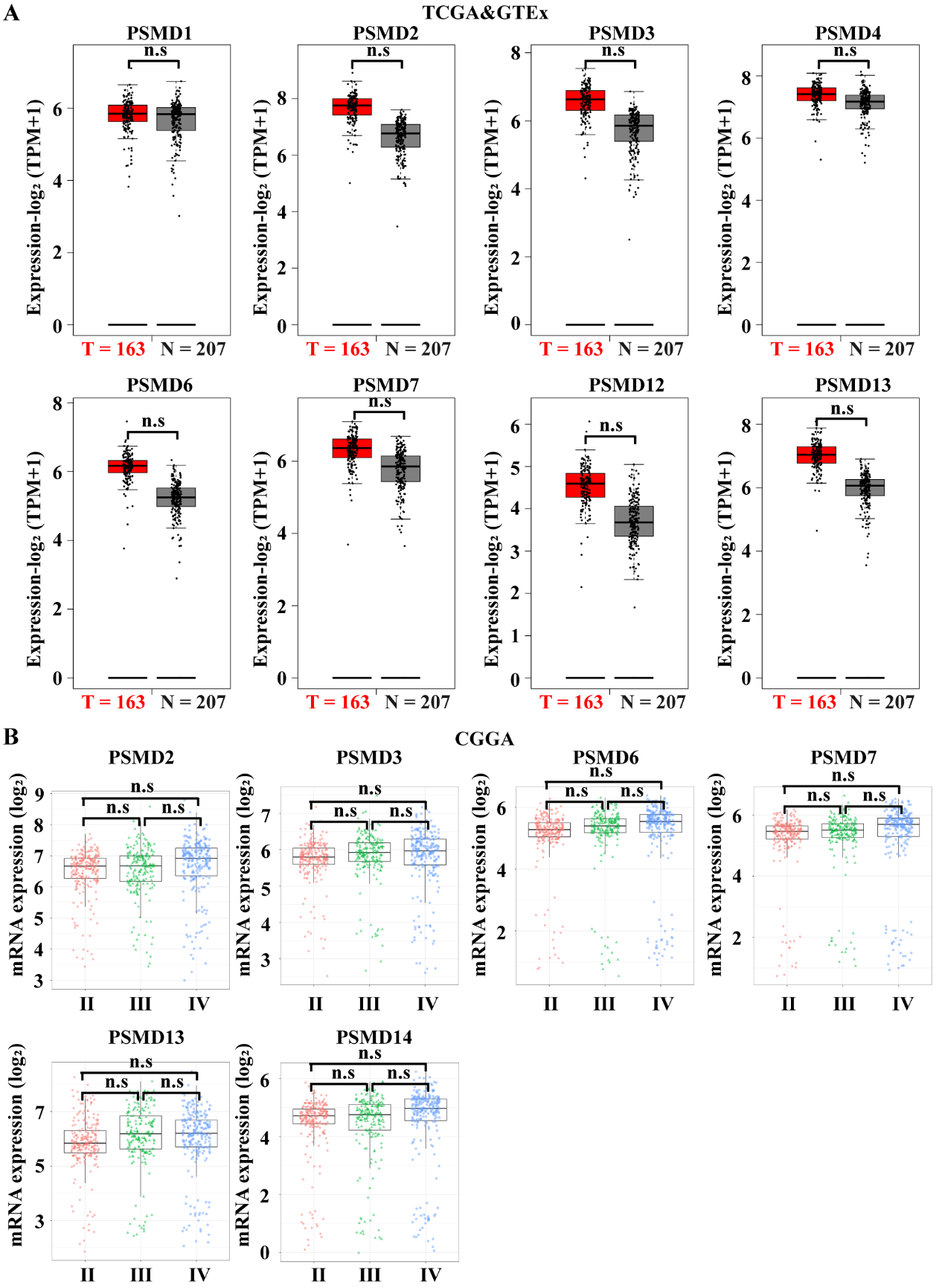


**Fig. S1 PSMD family mRNA expression levels in glioma.**

(A) mRNA expression levels of eight PSMD family members in GBM tissues and normal brain tissues from GEPIA2. T: GBM tissues; N: normal tissues. (B) Relationships between the mRNA expression levels of six PSMD family members and the grades of glioma from GlioVis. n.s=no significance.


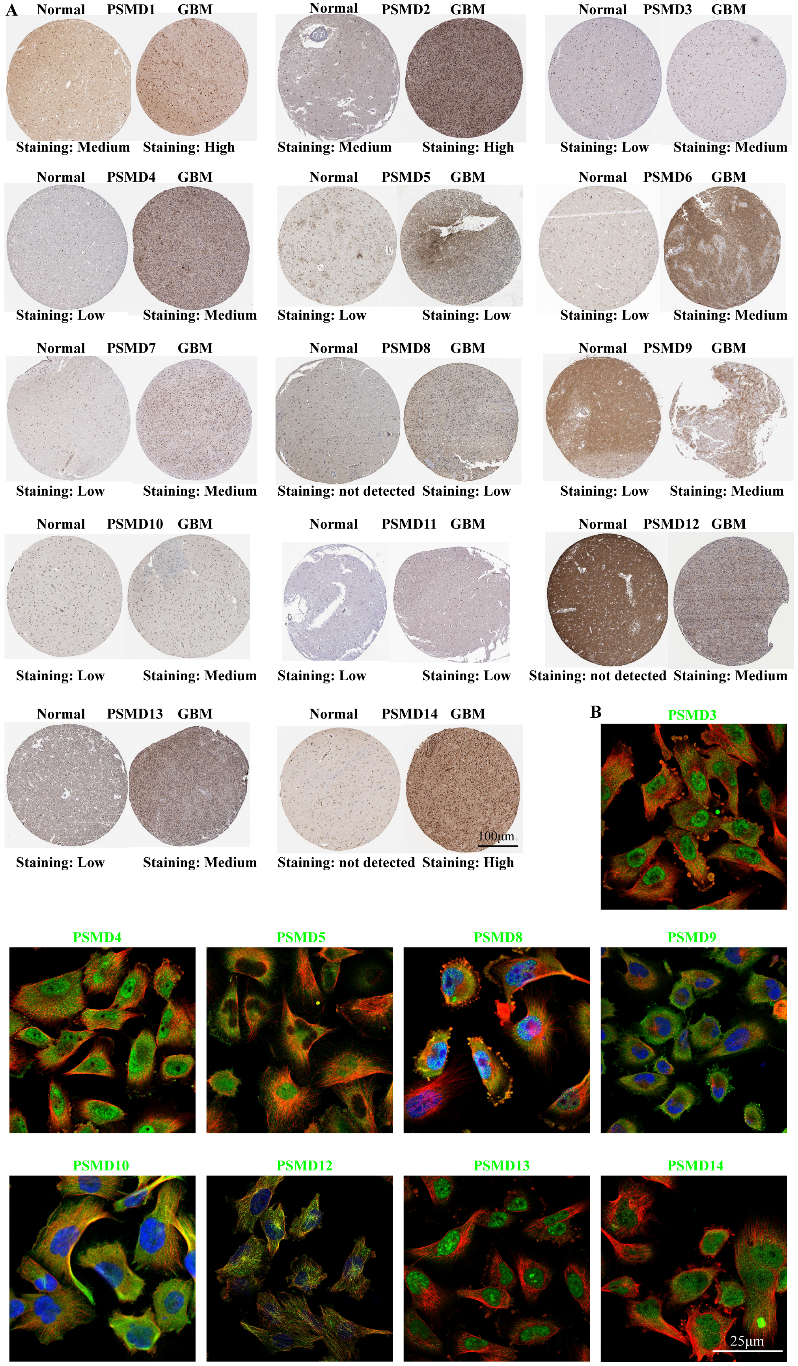


**Fig. S2 Representative immunohistochemistry (IHC) and immunocytochemistry images of PSMD family members staining from the Human Protein Atlas database.**

(A) The protein expression data of PSMD in GBM tissues and normal brain tissues. (B) Localization of PSMD in U251 GBM cell line: PSMD3: nucleoplasm and cytosol toggle channels; PSMD4: nucleoplasm and cytosol toggle channels; PSMD5: nucleoplasm and cytosol toggle channels; PSMD8: nuclear speckles and cytosol toggle channels; PSMD9: plasma membrane and cytosol toggle channels; PSMD10: cytosol; PSMD12: microtubules toggle channels; PSMD13: nucleoli and nuclear speckles toggle channels; PSMD14: nucleoplasm and cytosol toggle channels. Green represents the target protein, and red represents microtubules.


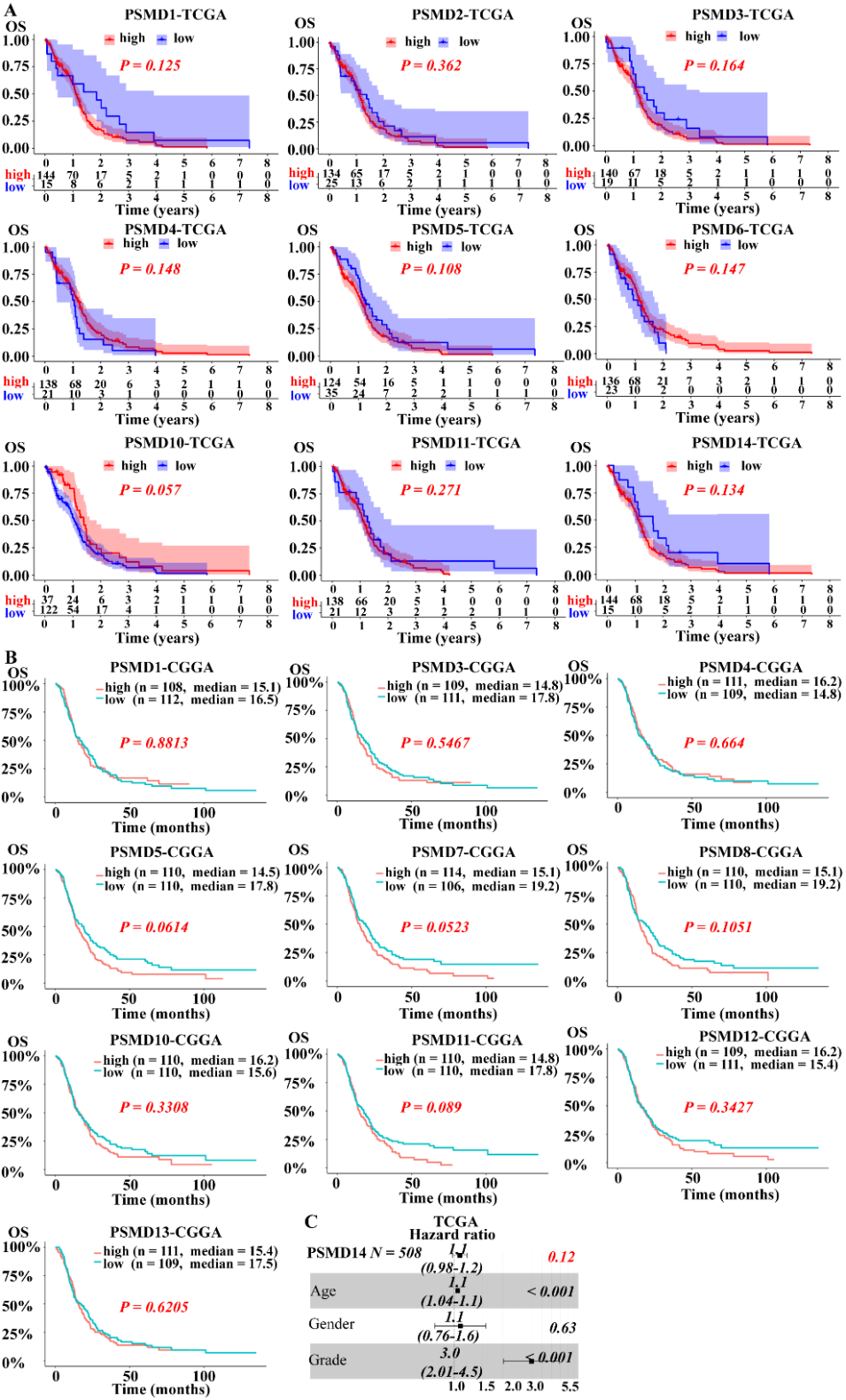


**Fig. S3** **Prognostic performance of PSMD expression in GBM patients.**

(A) Kaplan–Meier survival analyses of PSMD family members from the TCGA database. (B) Kaplan–Meier survival analyses of PSMD family from the CGGA database. (C) Multivariate Cox analysis of the ability of PSMD14 and other clinicopathological variables to predict the OS of GBM patients. The results are presented as the hazard ratio (HR). The bar represents the 95% confidence interval (CI) of the HR value. OS: overall survival.


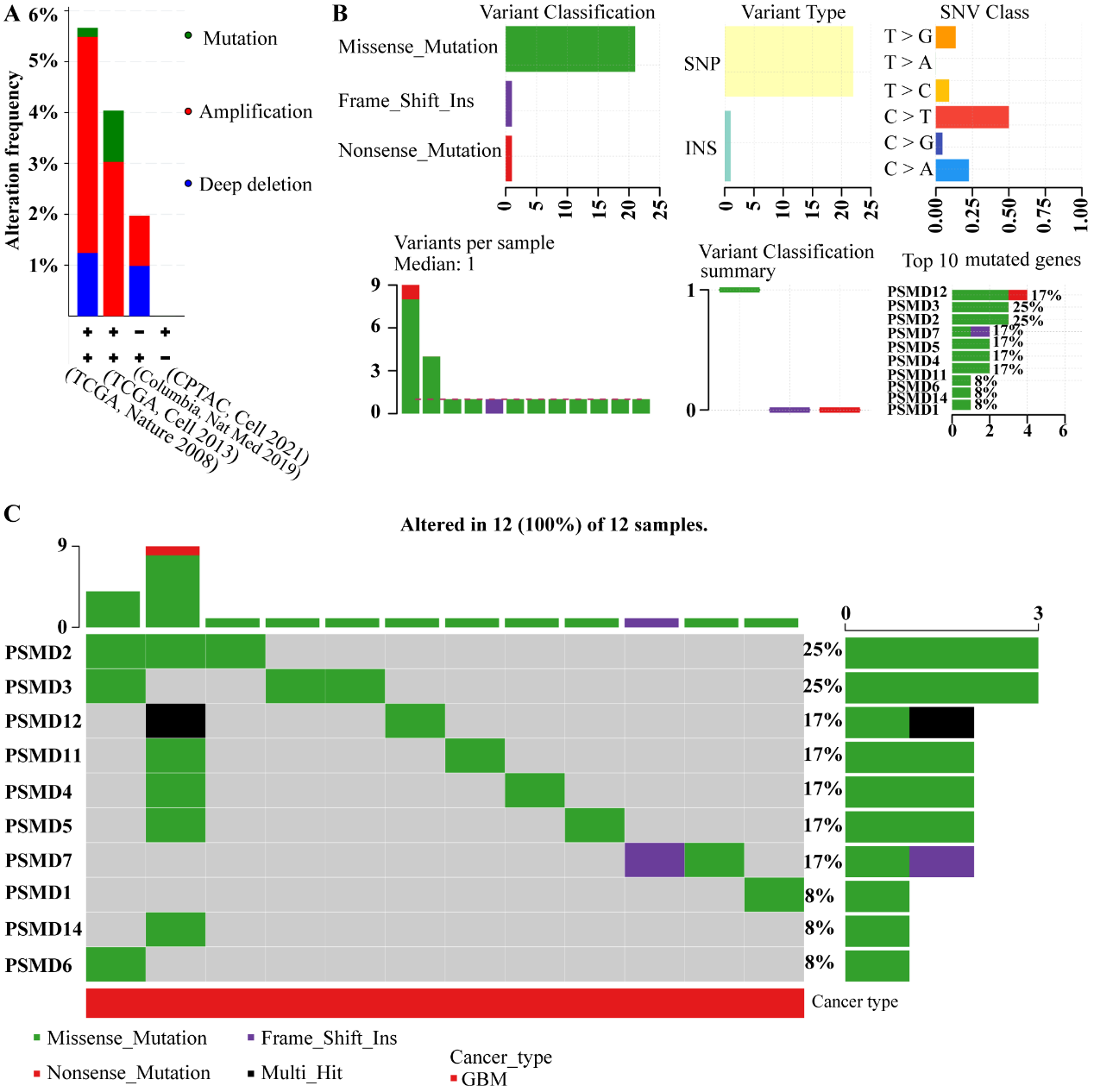


**Fig. S4 PSMD genetic alteration profiles in GBM.**

(A) PSMD alteration frequency in GBM. (B) Summary of the count for each type of effective mutation and SNP, as well as the count for each SNV class and variant. (C) Waterfall plot showing the genetic variation distribution of PSMD family genes in GBM and the classification of SNV types. SNV: single nucleotide variant, SNP: single nucleotide polymorphism.


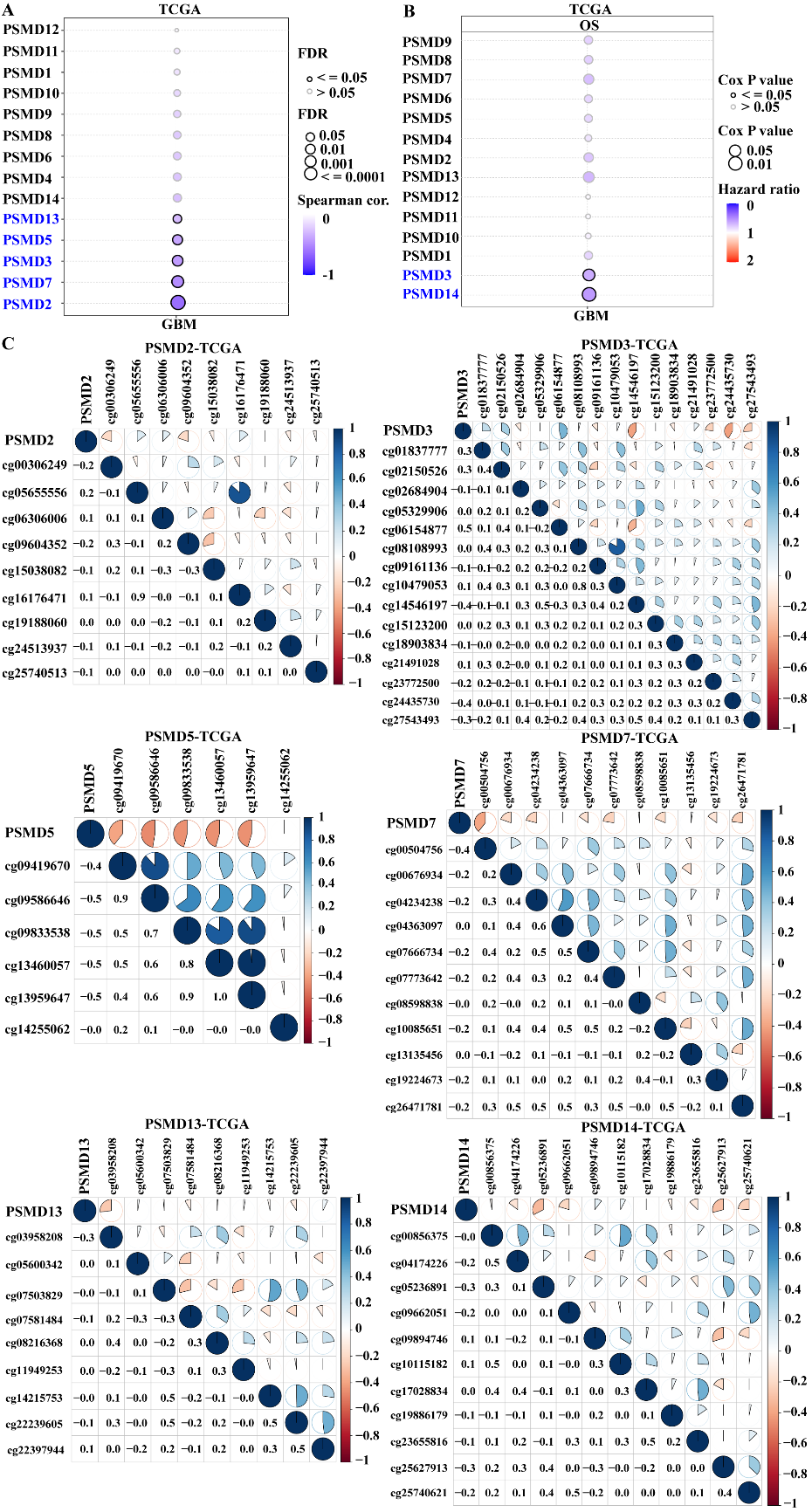


**Fig. S5 DNA methylation of the PSMD family.**

(A) Correlations between methylation and mRNA expression of PSMD family members from the GSCA. (B) OS differences between GBM patients with high and low methylation of PSMD from the GSCA. (C) Methylation sites that regulate PSMD2/3/5/7/13/14 mRNA expression from the TCGA. OS: Overall survival.


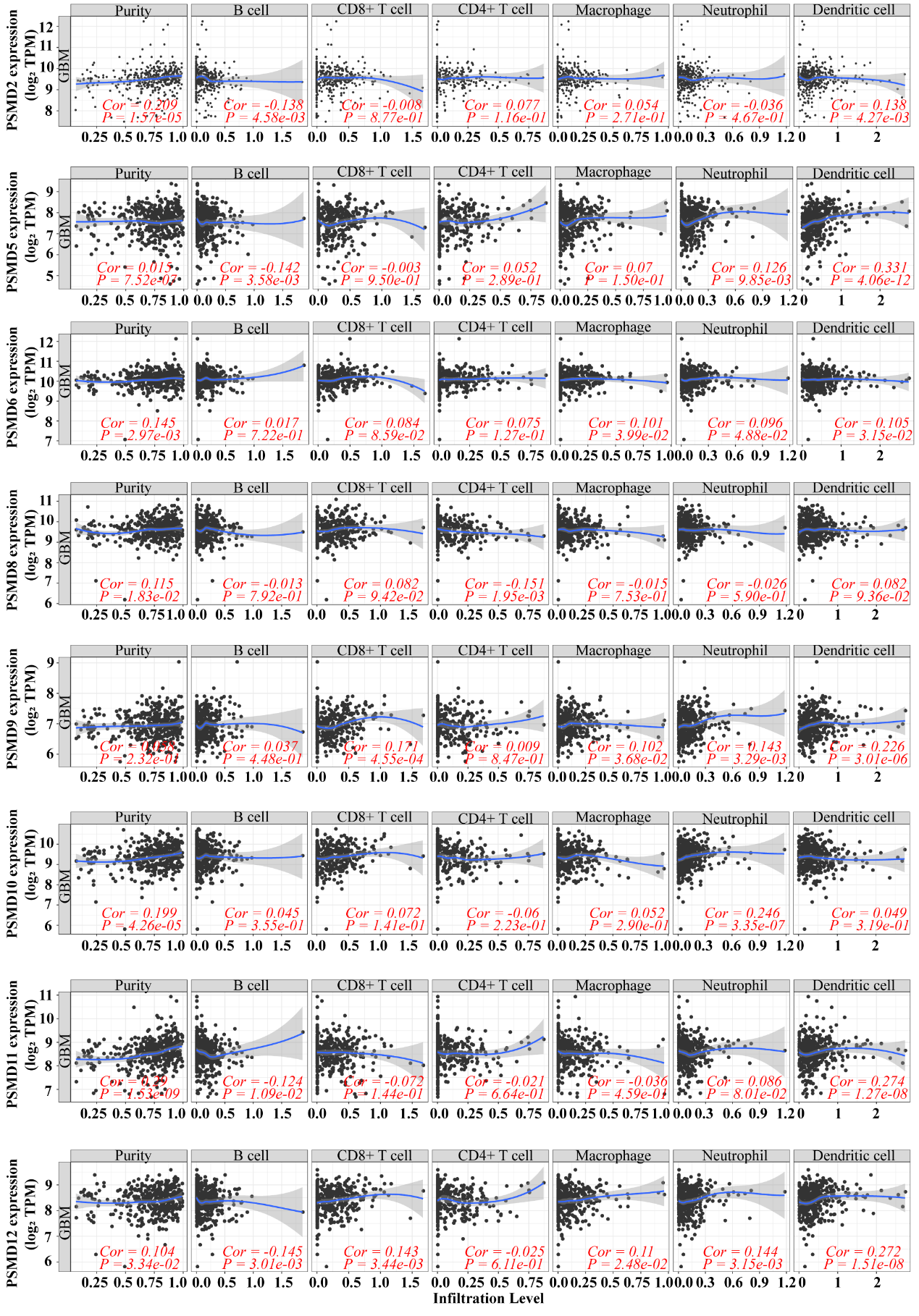


**Fig. S6 Correlations between PSMD expression and immune cell infiltration.**

Correlations between the expression of PSMD family members and the infiltration abundance of six immune cell types (TIMER2).


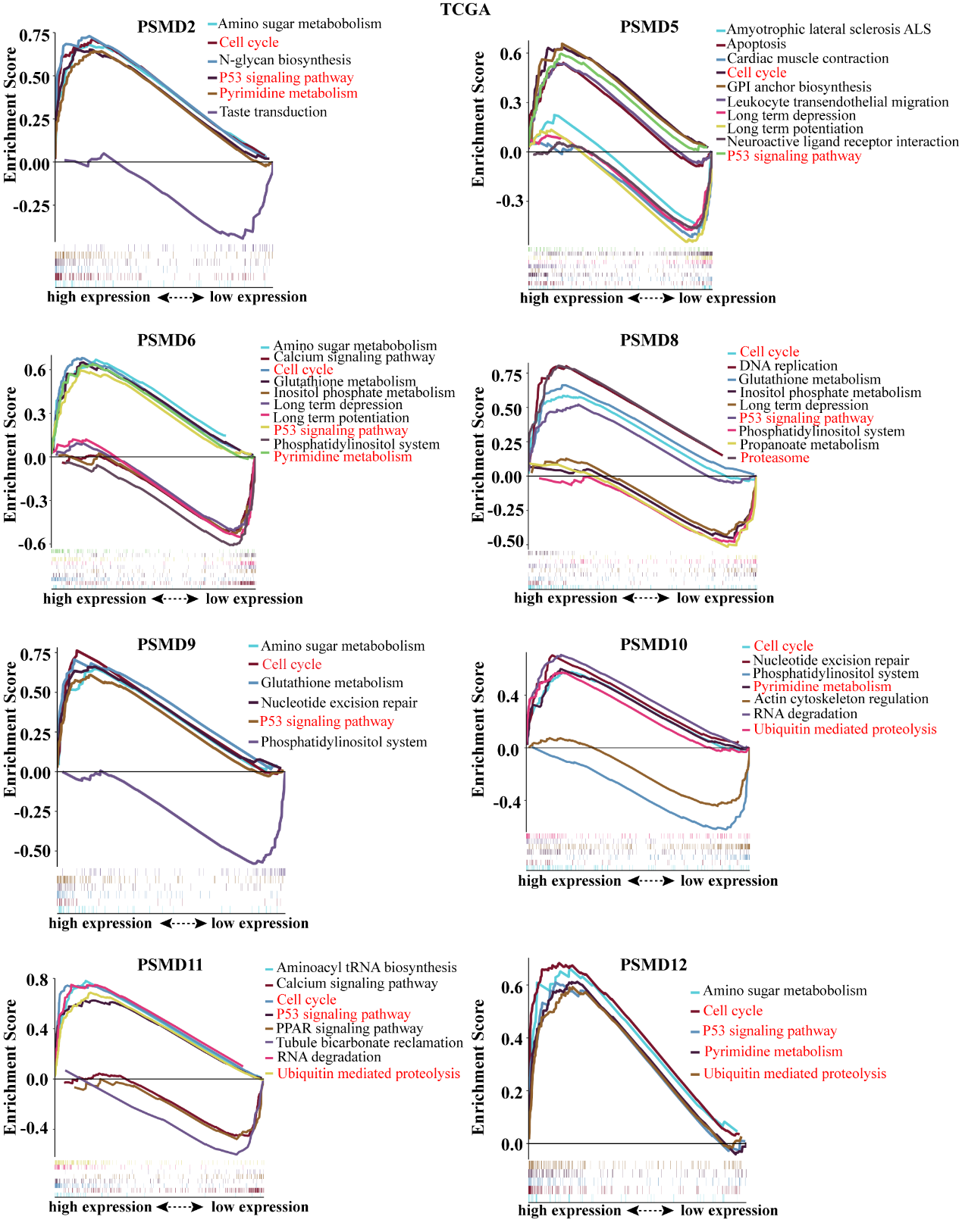


**Fig. S7 GSEA KEGG enrichment analysis results.**

GSEA results based on hallmark and KEGG datasets in GBM.


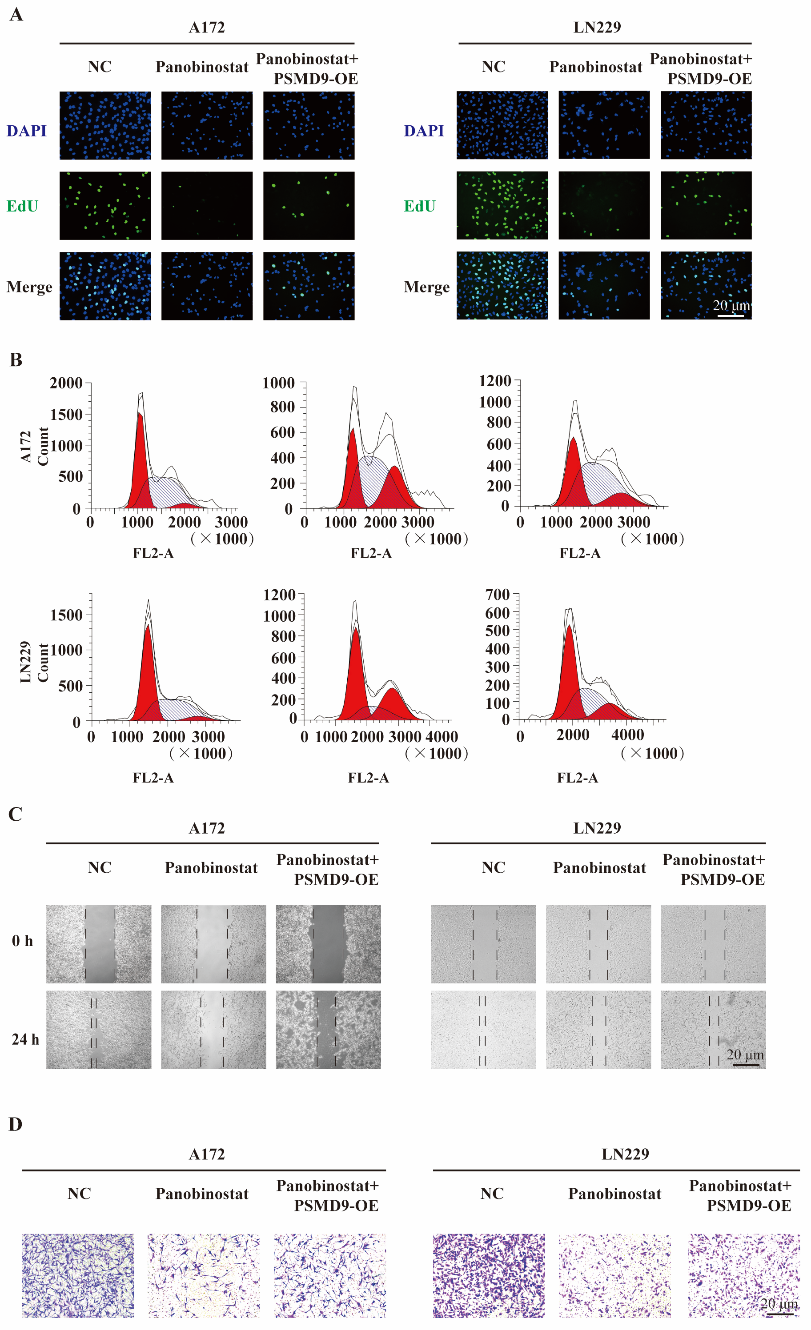


**Fig. S8 Overexpression (OE) of PSMD9 partially reversed the anti-glioblastoma effect of panobinostat.**

(A) Representative images of EdU assays for A172 and LN229 cells under the conditions indicated, with or without PSMD9-OE. Scale bar, 20 μm. (B) Flow cytometry to detect the percentage of G2/M phase of A172 and LN229 cells under the conditions indicated. (C) Representative images of wound healing assays for A172 and LN229 cells under the conditions indicated. Scale bar, 20 μm. (D) Representative images of transwell assays for A172 and LN229 cells under the conditions indicated, with or without PSMD9-OE. Scale bar, 20 μm.
