# Supplementary material for: Comprehensive analysis of PSMD family members and validation of PSMD9 as a potential therapeutic target in human glioblastoma
Source: CNS Neurosci Ther. 2023 Jul 23;30(2):e14366. doi: 10.1111/cns.14366 (PMC10848081; doi:10.1111/cns.14366)
Supplement: Supplementary file 2 — Appendix S1 [file CNS-30-e14366-s002.pdf]

Fig. 7D

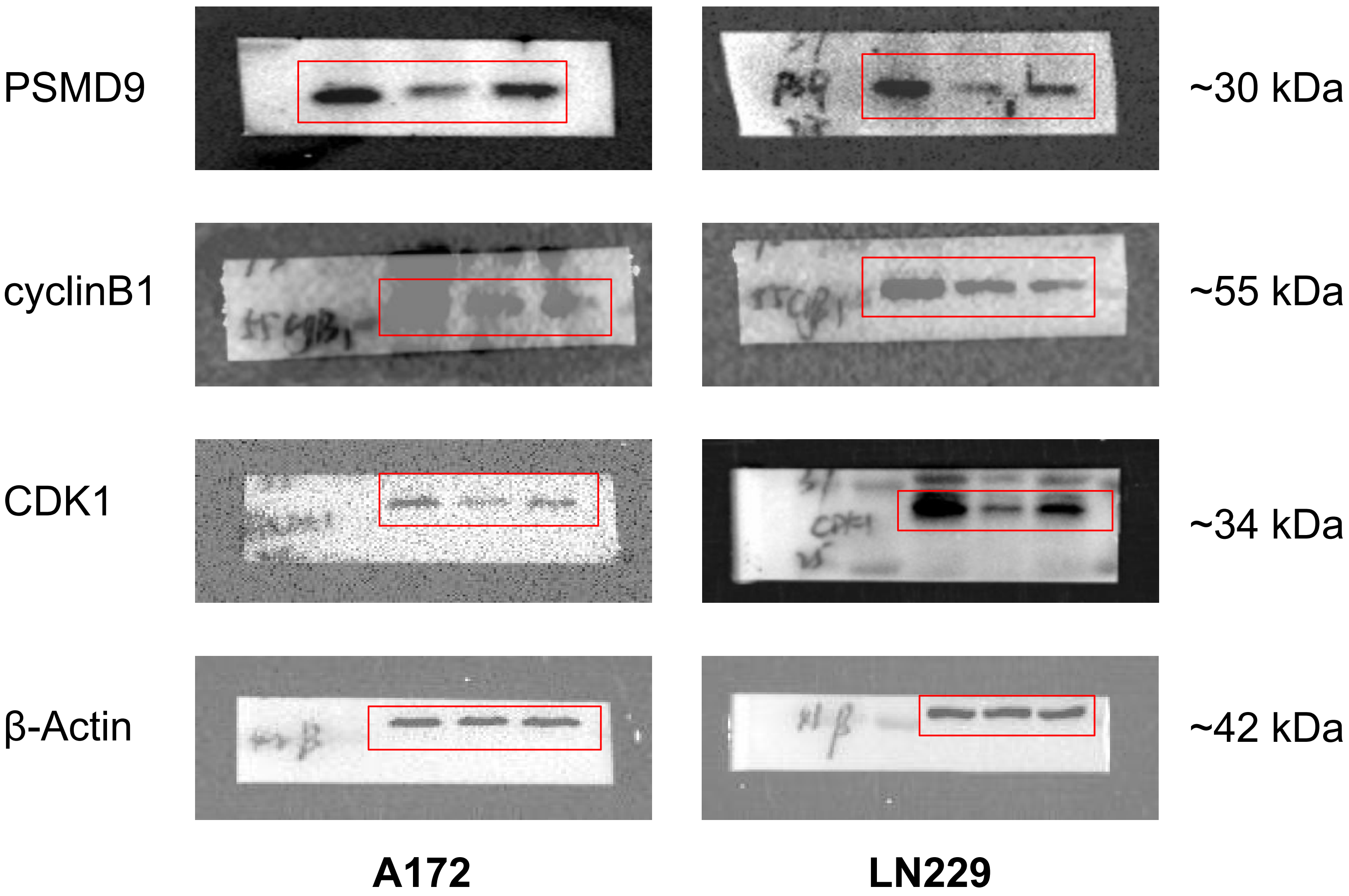
PSMD9
cyclinB1
CDK1
β-Actin
~30 kDa
~55 kDa
~34 kDa
~42 kDa
A172
LN229

Fig. 7E

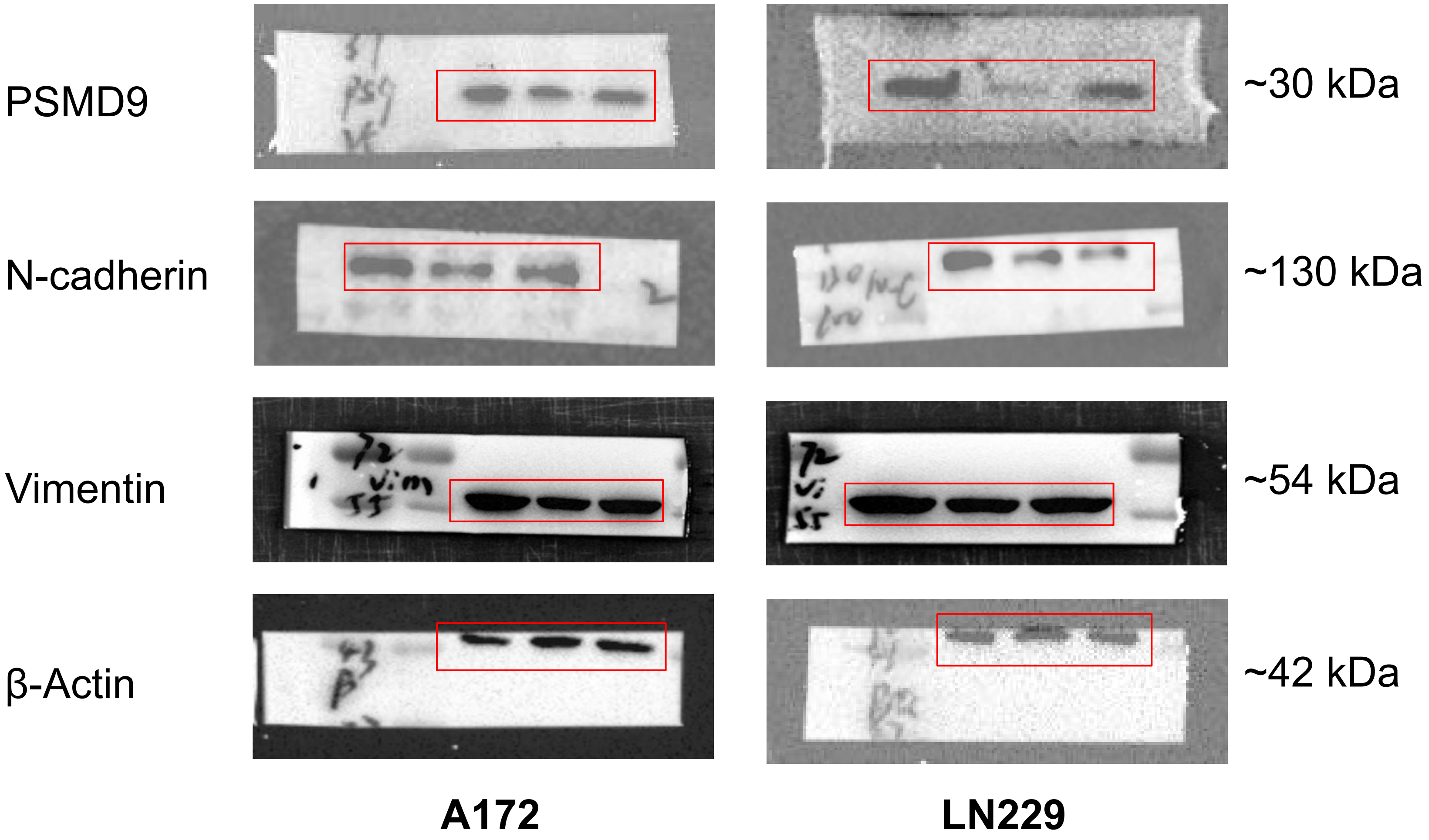
PSMD9
N-cadherin
Vimentin
β-Actin
~30 kDa
~130 kDa
~54 kDa
~42 kDa
A172
LN229

Fig. 8B

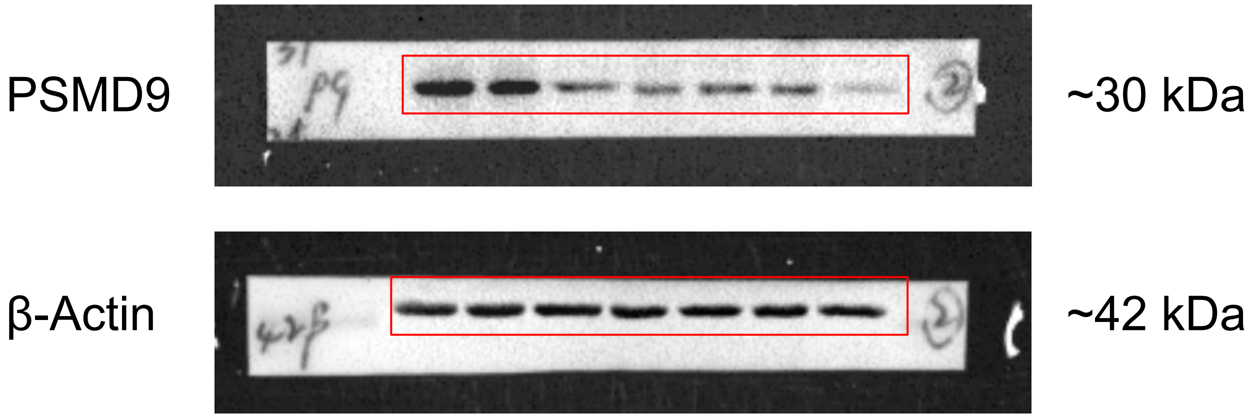

Fig. 9F

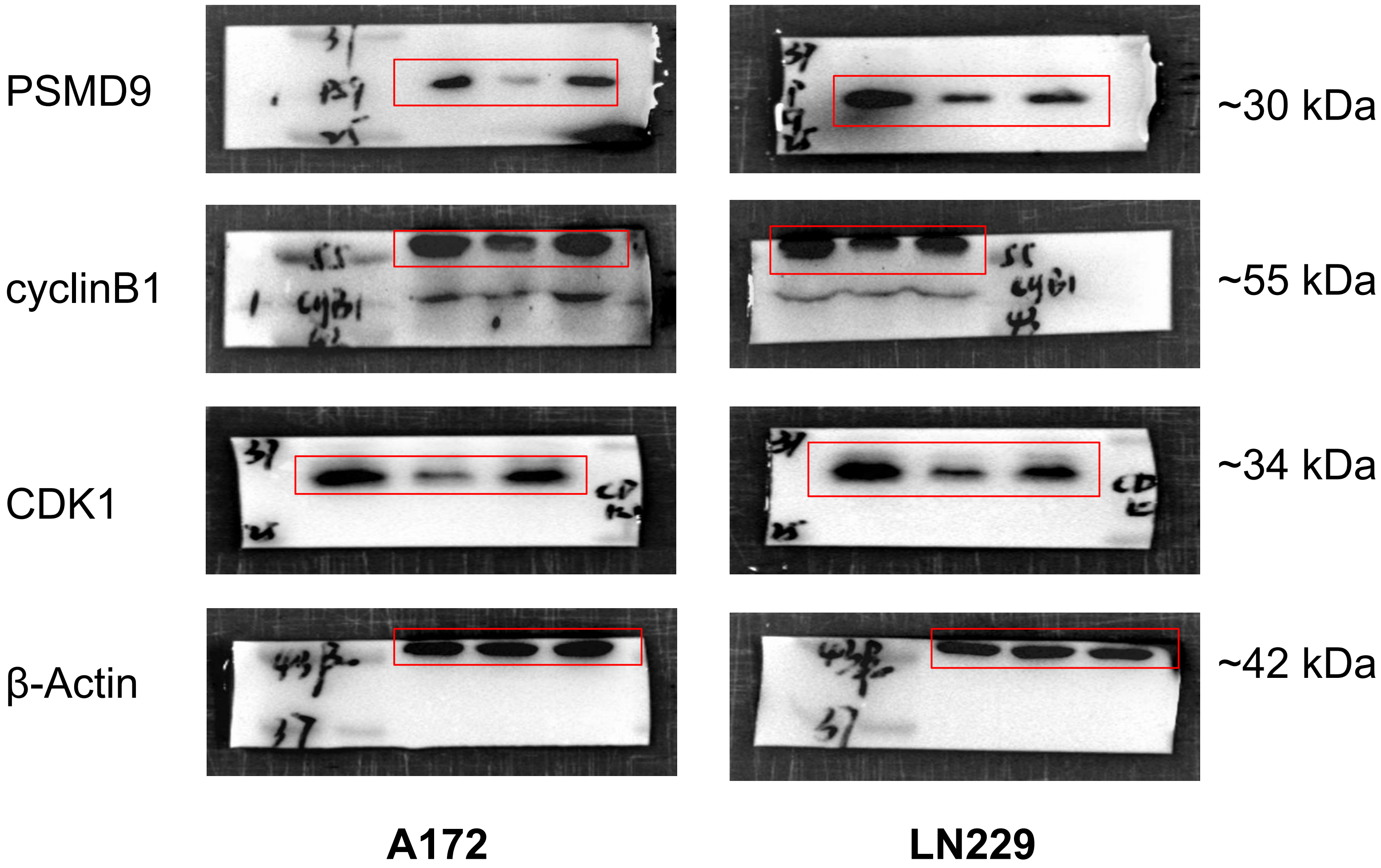
PSMD9
cyclinB1
CDK1
β-Actin
~30 kDa
~55 kDa
~34 kDa
~42 kDa
A172
LN229

Fig. 9G

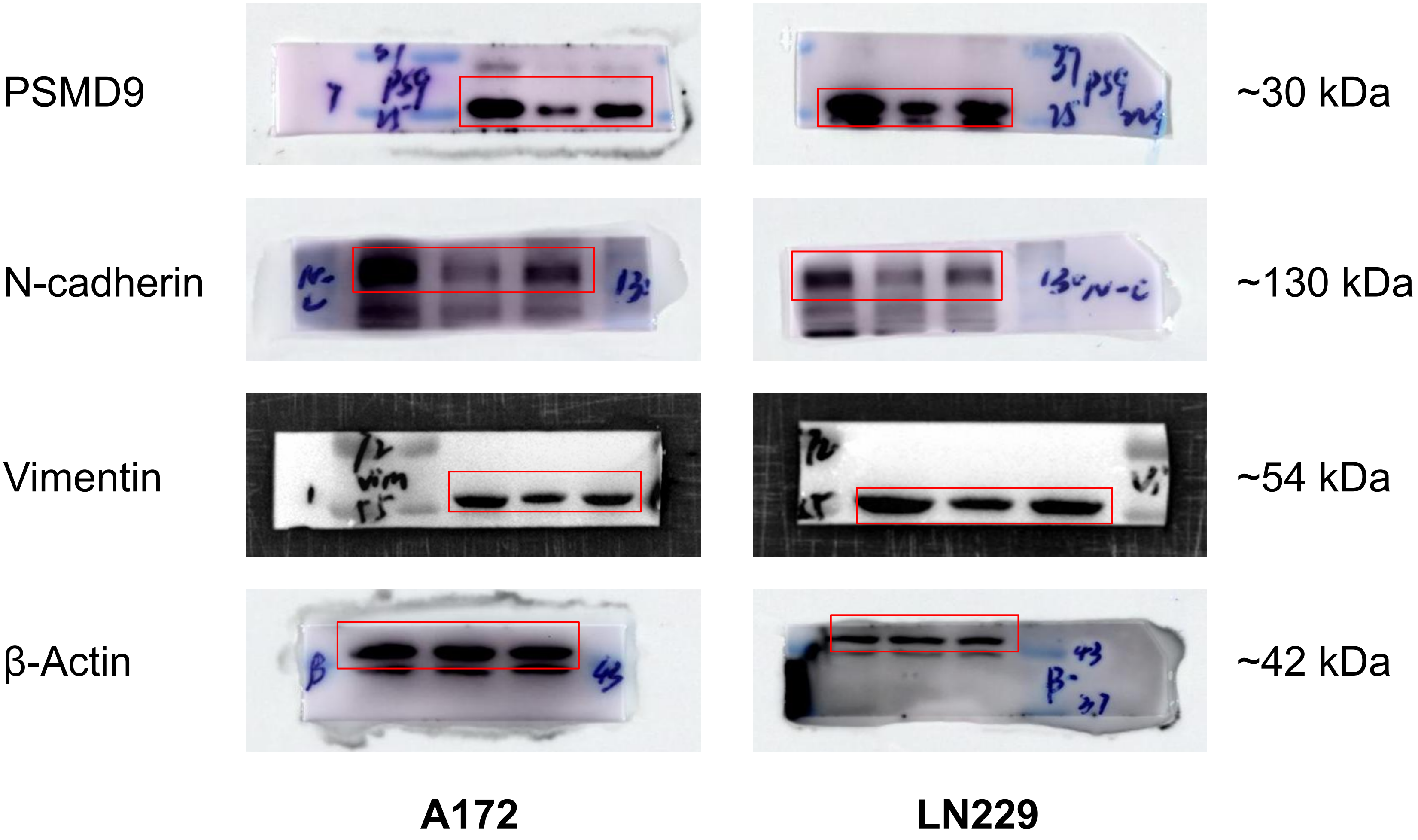
PSMD9
~30 kDa
N-cadherin
~130 kDa
Vimentin
~54 kDa
β-Actin
~42 kDa
A172
LN229
